# Strategies adopted by the nursing team for the prevention of pressure ulcers in hospitalized older people: a scoping review

**DOI:** 10.1590/1980-220X-REEUSP-2024-0362en

**Published:** 2025-11-21

**Authors:** Maria Clara Nascimento Oliveira, Antonio Rosa de Sousa, Esteffany Vaz Pierot, Winner Gomes Machado, Francisco Gilberto Fernandes Pereira, Ana Larissa Gomes Machado

**Affiliations:** 1 Universidade Federal do Piauí, Programa de Pós-Graduação em Enfermagem, Teresina, PI, Brazil.; 2 Universidade Federal do Ceará, Programa de Pós-Graduação em Enfermagem, Fortaleza, CE, Brazil.

**Keywords:** Health of the Elderly, Aged, Pressure Ulcer, Nursing, Nursing Care

## Abstract

**Objective:**

To map the strategies described in the literature that can be adopted by the nursing team to prevent pressure injuries in hospitalized older people.

**Method:**

Structured scoping review according to the JBI methodology, performed on June 17, 2024. The search for documents was carried out in eight databases and in a library of theses and dissertations, with the help of the application Rayyan for selecting studies.

**Results:**

Eleven studies were included in the review. Initial risk assessment, ongoing reassessment and monitoring, pressure redistribution, maintenance of skin integrity, treatment of pressure injuries, nutritional and fluid support, education and involvement of patients and families, training of the healthcare team, in addition to the implementation of standardized instruments and the use of technology, were identified as the main prevention strategies.

**Conclusion:**

The identified prevention strategies allow the nursing team to act quickly, using integrated interventions optimized by professional training, as well as to use standardized instruments and advanced technologies.

## INTRODUCTION

A Pressure Ulcer (PU) is a localized damage to the skin or underlying tissues, usually over bony prominences or associated with medical devices, resulting from prolonged pressure alone or combined with shear, influenced by intrinsic and extrinsic factors that compromise tissue resistance^(1)^. The overall prevalence is 12.8%, with stages I (43.5%) and II (28.0%) predominating, and the most affected sites are the sacrum (37.3%), heels (29.5%) and hip (7.8%)^(2)^. Sustained pressure on these areas leads to ischemia and tissue necrosis; friction, shear, and moisture aggravate the process, favoring skin rupture^(3)^.

The risk is greater in people with reduced mobility or sensitivity, such as bedridden or wheelchair-bound patients, especially in the older population, whose skin shows changes related to aging^(4)^. The forecast of 2.1 billion people aged 60 and over by 2050 highlights the magnitude of this challenge and the need for effective preventive policies and practices^(5,6,7)^. In the hospital environment, PU prevention is a shared responsibility, with emphasis on the role of nurses in direct care. However, knowledge and skills limitations still compromise preventive practice, reinforcing the need for professional development^(8)^. Evidence-based practice is essential for safe and effective clinical decisions, but faces barriers such as training gaps and difficulties in incorporating recommendations^(9)^.

Despite campaigns and technological advances, the incidence and impact of PUs in hospitalized older people remain alarming, with few significant advances in recent decades. This scenario highlights the urgent need to intensify efforts to raise awareness, prevent and treat these injuries, as well as greater investment in research and clinical practice to develop more effective strategies^(10)^.

In Brazil, regulations such as the Nursing Professional Practice Law (Law No. 7,498/1986), COFEN Resolution No. 564/2017, and the National Patient Safety Program (PNSP), established by Ministry of Health Ordinance No. 529/2013, establish clear guidelines for the work of nurses in the prevention and treatment of PUs. These standards reinforce the importance of safe and qualified practices to ensure the safety and quality of care for hospitalized older people^(11,12,13)^.

Despite these regulations and the mandatory Patient Safety Centers (*NSP*), Brazil faces significant challenges in preventing PU. Between 2014 and 2022, PUs accounted for 20.3% of adverse event notifications in the National Health Surveillance System (*SNVS*), totaling 223,378 cases. This number places PUs as the second most frequent type of adverse event in the country. During this period, 26,735 “never events” were reported, of which 72.21% were related to stage 3 PUs and 21.57% to stage 4 PUs, with 65 deaths directly attributed to these injuries^(14)^.

These data show that, despite regulatory efforts, regional inequalities, insufficient resources and gaps in professional training persist. Many hospital services have inadequate infrastructure, including a lack of pressure redistribution mattresses and essential devices, which compromises preventive practices. Furthermore, socioeconomic disparities and increased demand for hospitalizations among older adults are straining the health system, hindering the implementation of evidence- based strategies^(14)^.

To ensure the originality of this review, a search was carried out in the scientific literature, covering several databases. Although there are global guidelines and systematic reviews on PU prevention, these recommendations are broad and aimed at multidisciplinary teams or long-term care facilities. However, there are no reviews focused exclusively on nursing performance in the hospital context.

Therefore, this review seeks to map the strategies adopted by nursing and identify the most relevant preventive practices for this scenario. The selection of a scoping review allows for a comprehensive view, highlighting not only the consolidated evidence, but also the gaps in the literature on nursing’s role in preventing PU in hospitalized older people.

The relevance of this study lies in the systematization of preventive strategies for hospitalized older people, a particularly vulnerable group, with the potential to improve clinical practices and ensure more effective care. Furthermore, by mapping the studies carried out by nursing, the role of this profession in the production of knowledge and the implementation of preventive interventions is reinforced. As a protagonist in the continuum of care, nursing can use this evidence to foster training, influence institutional policies, and strengthen its autonomy in preventing PU. Therefore, this scoping review aims at mapping the strategies described in the literature that can be adopted by the nursing team to prevent pressure ulcers in hospitalized older people.

## METHOD

### Design of the Study

This is a scoping review conducted according to the JBI methodology for this type of study^(15)^, following the guidelines of *Preferred Reporting Items for Systematic Reviews and Meta-Analyses 2020* (PRISMA 2020)^(16)^. The review protocol was registered in the *Open Science Framework* (OSF) under the Digital Object Identifier (DOI): 10.17605/OSF.IO/WRVZ5. All methodological modifications made during the development of the protocol were documented in this version.

### Review Research Question

To formulate the research question, the PCC (Population, Concept and Context) strategy was used: Population (hospitalized older people), Concept (PU prevention strategies) and Context (nursing care for hospitalized older people). Thus, the question was formulated as follows: “What strategies described in the literature can be adopted by the nursing team to prevent PUs in hospitalized older people?”

### Eligibility Criteria

The inclusion and exclusion criteria were established based on the PCC method (Population, Concept and Context)^(15)^. The target population included hospitalized older people, excluding studies that addressed other age groups. The concept focused on PU prevention strategies, while studies that did not address this topic in elderly people were excluded. The context considered nursing care for hospitalized older people. Various sources of evidence were included, without restrictions on language, time frame or country of origin, including articles, dissertations and theses. Methodological studies for translation or validation of instruments, theoretical essays, and editorials were excluded.

### Research Strategy

To develop the search strategy for this scoping review, a preliminary search was carried out in the PubMed® and Web of Science™ databases, using Medline medical subject headings (MeSH), Health Sciences descriptors (DeCS), and the Emtree thesaurus. Reading the titles and abstracts of the texts found in this preliminary research allowed the identification of other keywords and synonymous terms, with the aim of expanding the search results and obtaining a more sensitive strategy for the second phase of data selection.

Thus, the databases included: PubMed® (*National Library of Medicine*, NLM); Web of Science™ Core Collection (*Clarivate Analytics*); Scopus® (Elsevier); and Embase® (Elsevier). The databases Nursing Database (BDENF), Latin American and Caribbean Literature on Health Sciences Information (LILACS) and *Bibliographical Index Spanish in Health Sciences* (IBECS) were accessed through the Virtual Health Library (VHL). To include gray literature studies, the Brazilian Digital Library of Theses and Dissertations (*BDTD*) of the Coordination for the Improvement of Higher Education Personnel (CAPES) was also used.

The terms related to the PCC acronym were adapted for each data platform, taking into account the variations and Boolean operators (AND and OR) to develop the final strategies, presented in Chart 1.

**Chart 1:** Construction syntax, descriptors/keywords and Boolean operators used in databases – Teresina, PI, Brazil, 2024.

Database	Search Strategy
PubMed^®^ via NLM N = 1210	((“health of the elderly”[All Fields]) OR (“aged”[MeSH Terms]) OR (“health services for the aged”[MeSH Terms]) OR (“aged, 80 and over”[MeSH Terms]) OR (“hospitalization”[MeSH Terms])) AND ((“pressure ulcer”[MeSH Terms]) OR (“risk assessment”[MeSH Terms]))) AND ((“nursing”[MeSH Terms]) OR (“nursing, team”[MeSH Terms]) OR (“nursing care”[MeSH Terms]) OR (“nurses improving care for health system elders”[MeSH Terms]))
Web of Science™ Main Collection N = 1068	ALL = ((Aged* OR “Health of the Elderly” OR “Health Services for the Aged” OR “Health of the Elderly” OR “Aged, 80 and over” OR Hospitalization*) AND (Pressure Ulcer* OR “Risk Assessment”) AND (Nursing* OR Nursing, Team* OR “Nursing Care” OR “Nurses Improving Care for Health System Elders”))
Scopus^®^ N = 622	((Aged* OR “Health of the Elderly” OR “Health Services for the Aged” OR “Health of the Elderly” OR “Aged, 80 and over” OR Hospitalization*) AND (Pressure Ulcer* OR “Risk Assessment”) AND (Nursing* OR Nursing, Team* OR “Nursing Care” OR “Nurses Improving Care for Health System Elders”))
Embase^®^ N = 1688	(aged* OR ‘health services for the aged’/exp OR ‘health services for the aged’ OR ‘health of the elderly’ OR ‘aged, 80 and over’/exp OR ‘aged, 80 and over’ OR hospitalization*) AND ((‘pressure’/exp OR pressure) AND ulcer* OR ‘risk assessment’/exp OR ‘risk assessment’) AND ((nursing* OR ‘nursing,’/exp OR nursing,) AND team* OR ‘nursing care’/exp OR ‘nursing care’ OR ‘nurses improving care for health system elders’/exp OR ‘nurses improving care for health system elders’) AND [embase]/lim
BDENF, LILACS and IBECS via VHL N = 278	((“Saúde do Idoso”) OR (idos*) OR (“Serviços de Saúde para Idosos”) OR (“Saúde do Idoso”) OR (“Idoso de 80 Anos ou mais”) OR (Hospitalizaç*)) AND ((“Úlcera por Pressão”) OR (“Medição de Risco”)) AND ((“Enfermagem”) OR (“Equipe de Enfermagem”) OR (“Cuidados de Enfermagem”) OR (“Cuidado de Enfermagem ao Idoso Hospitalizado”))
CAPES BDTD N = 50	(“Saúde do Idoso” OR idoso OR “Serviços de Saúde para Idosos” OR “Saúde do Idoso” OR “Idoso de 80 Anos ou mais” OR Hospitalização) AND (“Úlcera por Pressão” OR “Medição de Risco”) AND (“Enfermagem” OR “Equipe de Enfermagem” OR “Cuidados de Enfermagem” OR “Cuidado de Enfermagem ao Idoso Hospitalizado”)

The searches were carried out on June 17, 2024, using remote access to databases through the journal portal of the Coordination for the Improvement of Higher Education Personnel (CAPES), accessed via the Federated Academic Community (CAFe) with the login of the Universidade Federal do Piauí (UFPI).

### Evidence Source Selection

The selection of material related to the theme took place with the help of the reference management system *Rayyan CQRI Systems* ^(17)^. Initially, files containing the literature found in each information source were exported to the system. Two reviewers independently excluded duplicate material. They then proceeded to read the summaries of the remaining texts, assigning acceptance or rejection concepts according to the established inclusion and exclusion criteria. The process was conducted blindly, using the resource *blind on* made available by the system, and any discrepancies between reviewers were resolved by a third reviewer.

After this stage, the selected texts were read in full and evaluated according to the inclusion and exclusion criteria. The accepted texts were used for data extraction. The results of this process were organized in a diagram that presents the phases of identification, screening, inclusion, and selection of texts for review.

### Data Extraction

Data extraction for inclusion in this review was performed based on the list of references generated in Rayyan, which was exported to a Microsoft Excel spreadsheet. Subsequently, the three reviewers completed the spreadsheet independently, following a form designed specifically for this stage, with the aim of meeting the purpose and question of the review. During the protocol phase, a draft table was developed and tested to record the main information. This draft was refined throughout the review phase and resulted in the final version, which included the following data extracted from each source: source of information, author(s), title, type, method, country, language, descriptors/keywords, and PU prevention strategies in older adults implemented by the nursing team.

### Presentation of Results

The findings were summarized using the data reduction method, through critical reading and classification of the results into conceptual categories^(18)^. The results were presented in figures and charts. The first chart detailed the descriptive information of the studies, including the source of information, author(s), title, type, method, country, language and descriptors/keywords. The second chart summarized the PU prevention strategies in hospitalized older people that can be applied by the nursing team.

**Figure 1:**
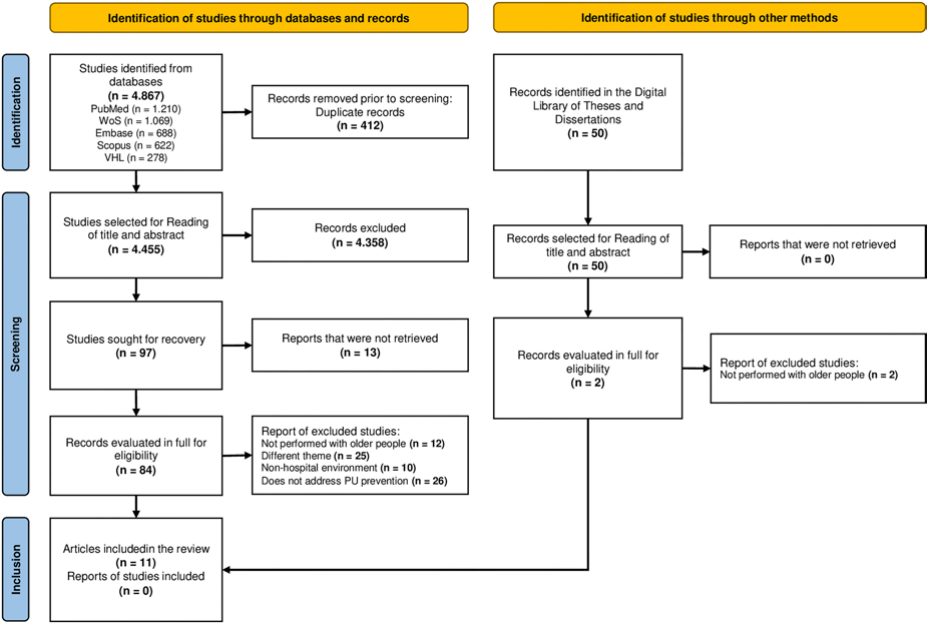
Flowchart of the process of identification, screening, and inclusion of articles – Teresina, PI, Brazil, 2024.

Source: Adapted from Page *et al.* (2021)^(16)^.

**Figure 2:**
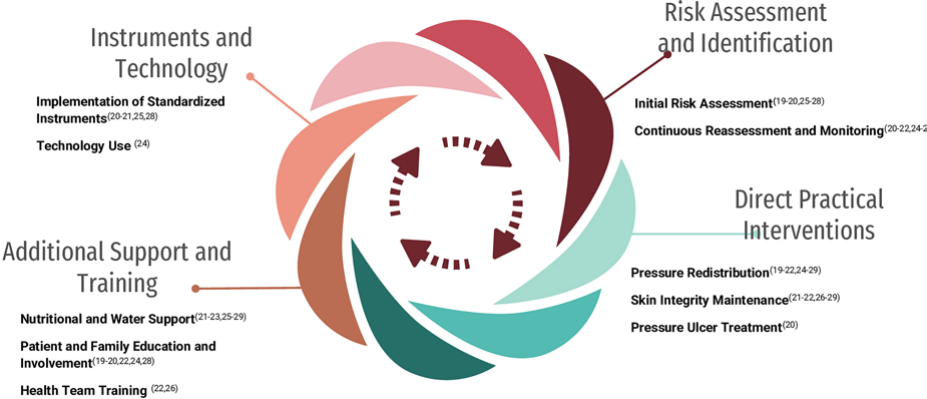
Circular diagram of pressure injury prevention strategies in older people carried out by the nursing team – Teresina, PI, Brazil, 2024.

Figure 1 presents the process of identifying, screening, and including articles. Figure 2, a circular diagram, organizes the PU prevention strategies in hospitalized older adults identified in the review. Unlike a hierarchy, the diagram displays strategies interdependently, with interventions positioned in segments that highlight their complementarity and equal relevance. This format favors an integrated visualization of actions, highlighting the contribution of each strategy to the effective prevention of PU in the context of hospitalized older people.

The discussion was based on selected literature and other scientific studies related to the topic. Furthermore, documents from entities that promote the creation and dissemination of evidence-based guidelines were used, with the aim of educating and training health professionals, in addition to developing policies aimed at improving the prevention and treatment of PUs.

### Ethical Aspects

As this was a scoping review without the direct involvement of human participants, there was no need for submission to the Research Ethics Committee, as per JBI guidelines. All stages were conducted in accordance with the ethical principles applicable to research, ensuring transparency, methodological rigor, and scientific integrity. Copyright and correct citation of sources were respected, ensuring the reproducibility and reliability of the findings.

## RESULTS

The search strategies identified a total of 4,867 publications. After removing 412 duplicates, 4,455 studies remained for screening titles and abstracts. Of these, 97 were selected for full reading. During this phase, 13 studies could not be retrieved, resulting in 84 articles that were read in full. After a thorough analysis, 73 articles were excluded, and 11 studies remained to compose this review. All reasons for exclusion were documented to create a report of those excluded for later presentation. It is important to highlight that it was not possible to identify additional studies using other methods, thus not adding further evidence to the present review (Figure 1).

From the analysis of the 11 studies included, it was found that the journal with the largest number of publications on the topic was *International Wound Journal*, with two publications (18.18%). Furthermore, the studies were carried out by 36 different authors. The years with the highest number of publications were 2021, 2019 and 2015, each with two publications (18.18%), while the other years recorded one publication (9.09%) each. Regarding typology, it was found that all the studies analyzed were scientific articles, nine of which were field studies (81.82%). The most used method was the quasi-experimental method, present in three studies (27.27%). Furthermore, China was the most representative country of origin, with three studies (27.27%).

Regarding language, most texts were available in English (72.73%). Finally, the keywords highlighted strategies for preventing and treating PU in older adults, emphasizing the importance of continuous nursing care, nutritional therapy, and the use of technology for real-time feedback. This information is detailed in Chart 2.

**Chart 2:** Selected information sources – Teresina, PI, Brazil, 2024.

No.	Source and year	Authors	Title	Country/language	Keywords	Method	Results
01	*Int. Wound J.* 2024	Deng et al.^(19)^	*Effects of predictive nursing interventions on pressure ulcer in elderly bedridden patients*	China/English	*Bedridden; Efficacy; Elderly;* *Predictive Nursing; Pressure Ulcers*	Quasi-experimental, comparing control group (conventional care) and intervention group (predictive nursing), n = 120.	The incidence of PU was significantly lower in the intervention group (10%) compared to the control group (38.3%) (p < 0.001). Braden scale scores were higher in the intervention group (p < 0.001). Time to onset of PU was longer in the intervention group (14.57 ± 3.21 days vs. 6.25 ± 2.36 days, p < 0.001). Anxiety (SAS) and depression (SDS) scores were lower in the intervention group after care (p < 0.001). Patient satisfaction was higher in the intervention group (98.3% vs. 85%, p = 0.008).
02	*Am. J. Transl. Res.* 2021	Xiao et al.^(20)^	*The preventive effect of seamless nursing care on pressure ulcer and related complications in elderly inpatients*	China/English	*Seamless Nursing Care; Elderly Inpatients; Pressure Ulcer; Complications*	Randomized clinical trial with 132 hospitalized older people (≥65 years), divided into a control group (conventional care, n = 66) and an intervention group (conventional care + continuous nursing, n = 66).	The incidence of PU was lower in the intervention group (3.03%) compared to the control group (21.21%) (p < 0.001). The complication rate was lower in the intervention group (9.09% vs. 33.33%, p < 0.05). Mean length of hospital stay was reduced in the intervention group (21.34 ± 4.38 vs. 26.24 ± 6.11 days, p < 0.001). Patient satisfaction was higher in the intervention group (83.33% vs. 59.09%, p = 0.002).
03	*Rev. Esc. Enferm.* 2021	Morrudo Garcia et al.^(21)^	Diagnóstico de enfermagem em pessoa idosa com risco para lesão por pressão	Brazil/Portuguese	*Idoso; Hospitalização; Lesão por Pressão; Diagnóstico de Enfermagem; Cuidados de Enfermagem*	Exploratory, descriptive, cross-sectional, quantitative study, carried out with 87 medical records of older people hospitalized in a medical clinic unit of a university hospital in Brazil.	24 older people (27.6%) were at risk for PU. Associated factors: being bedridden, restricted positioning in bed, and need for assistance with mobility (p < 0.05). Predominant interventions: mobility, pressure control, skin monitoring, nutrition and hygiene.
04	*Int. J. Clin. Exp. Med.* 2020	Yu et al.^(22)^	*Analysis of the effect of comprehensive nursing on pressure ulcers risk and psychological state in severe elderly patients*	China/English	*Pressure Ulcers; Elderly; Severe; Psychological State; Comprehensive Nursing*	Retrospective study comparing 103 critically ill older women before (control group, 2017) and 103 after (intervention group, 2018) the implementation of comprehensive nursing care.	The incidence of PU was significantly lower in the intervention group (11.65%) compared to the control group (39.81%) (p < 0.05). The mean time for PU formation was shorter in the intervention group (4.53 ± 2.81 days vs. 10.81 ± 3.56 days, p < 0.05). Patient satisfaction was higher in the intervention group (91.14% vs. 76.67%, p < 0.05).
05	*Int. Wound J.* 2019	Eglseer et al.^(23)^	*Nutritional management of older hospitalised patients with pressure injuries*	Austria/English	*Hospital; Malnutrition; Nutrition Therapy; Pressure Injury; Pressure Ulcer*	Multicenter cross-sectional study in 33 hospitals in Austria with 1412 hospitalized patients ≥70 years old. It evaluated nutritional interventions, risk of PU and nutritional status.	48% of patients were at risk of developing PU and 5% had PU. Most frequent nutritional interventions: feeding support (50.7%), nutritional screening (39.4%). Only 28.2% of patients with PU were referred to a nutritionist.
06	*J. Wound Ostomy Continence Nurs.* 2019	Hultin et al.^(24)^	*Information and communication technology can increase patient participation in pressure injury prevention: a qualitative study in older orthopedic patients*	Sweden/English	*Information and Communication Technology; OlderPatients; Patient Participation; Pressure Injuries; Pressure Injury Prevention; Real-Time Feedback*	Qualitative study in an orthopedic rehabilitation unit in Sweden with 31 patients ≥65 years old. Data collected through semi-structured interviews analyzed using content analysis.	Most patients (n = 21) were able to use the *Continuous Bedside Pressure Mapping* (CBPM). Participants reported greater knowledge about pressure ulcer (PU) prevention and began repositioning themselves more frequently. The main benefits were increased independence in self-care and active participation in PU prevention. CBPM provided real-time visual feedback, allowing immediate adjustments to patients’ position to reduce pressure on vulnerable areas. The technology has been found to be useful in preventing PU and can be integrated into clinical care as a standard feature.
07	*Appl. Nurs. Res.* 2016	Bååth et al.^(25)^	*Prevention of heel pressure ulcers among older patients – from ambulance care to hospital discharge: A multi-centre randomized controlled trial*	Sweden/English	*Heel Pressure Ulcer; Prevention; Ambulance Care; Emergency Department; Acute Care Delivery Chain; RCT*	Multicenter randomized clinical trial with 183 older people (≥70 years) transported by ambulance. Intervention group used heel suspension device.	The incidence of heel PU was significantly lower in the intervention group (14.6%) compared to the control group (30%) (p = 0.017). Less pain associated with injuries was reported in the intervention group. The heel suspension device reduced the time of direct skin contact with support surfaces, minimizing PU formation. The results suggest that the use of technological devices, such as pressure redistribution supports, should be routinely incorporated into pre-hospital care.
08	*Nurs. Stand.* 2015	Barry and Nugent^(26)^	*Pressure ulcer prevention in frail older people*	Ireland/English	*Education; Frail Older People; Incidence; Older People; Pressure Ulcers;* *Pressure Ulcer Prevention; Prevalence; Wound Care; Wound Management*	Narrative review on PU prevention in frail older people, addressing educational programs, clinical leadership, and multidisciplinary work.	Structured educational programs reduced the incidence of PU in frail older people by 73%. The SSKIN (Surface, Skin Inspection, Keep Moving, Incontinence, Nutrition) approach has demonstrated effectiveness in prevention and standardization of care. The study emphasizes the need to use pressure monitoring technologies and specialized surfaces to optimize PU prevention.
09	*Index Enferm.* 2015	Sousa et al.^(27)^	*Concepciones teóricas de Neuman asociadas con la prevención de las úlceras por presión: Un estudio de caso*	Brazil/Spanish	*Úlcera por Presión; Teoría de Enfermería; Cuidado de Enfermería*	Case report based on Betty Neuman’s Systems Theory for PU prevention. Case of an older woman hospitalized for 64 days in the Intensive Care Unit (ICU).	The patient did not develop PU during hospitalization, suggesting the effectiveness of preventive strategies based on Neuman’s Theory.
10	*Crit. Care Nurs. Clin. North Am.* 2007	Stotts and Wu^(28)^	*Hospital recovery is facilitated by prevention of pressure ulcers in older adults*	USA/English	No keywords	Narrative review on PU prevention in hospitalized older people and impact on recovery.	The incidence of PU in hospitalized older people varies between 0.4% and 38%. Prevention is associated with shorter hospital stays and better functional outcomes.
11	*Geriatr. Nurs.* 1997	Maklebust^(29)^	*Pressure ulcers: decreasing the risk for older adults*	USA/English	No keywords	Narrative review on risk factors and prevention strategies for PU in older people.	Major risk factors include immobility, excessive moisture, friction, shear, and malnutrition. Preventive measures include repositioning, support surfaces, and adequate nutrition.

The analysis of strategies for preventing PU in hospitalized older people revealed 10 important approaches that can be carried out by the nursing team: initial risk assessment; continuous reassessment and monitoring; pressure redistribution; maintenance of skin integrity; PU treatment, nutritional and fluid support; education and involvement of patients and families; training of the health team, implementation of standardized instruments and use of technology (Chart 3).

**Chart 3:** Strategies for preventing pressure ulcers in older adults that can be performed by the nursing team – Teresina, PI, Brazil, 2024.

No.	Strategy	Description
01	Initial Risk Assessment	Perform an initial assessment with risk scales, such as Braden and Norton, to identify patients at high risk for PU. Regularly document and update the assessment to reflect changes in the patient’s condition^(19,20,25–28)^.
02	Continuous Reassessment and Monitoring	Inspect the skin daily for early signs of PU, such as changes in color or integrity. Document the results, implement immediate interventions when necessary, and adjust the care plan based on observations^(20-22,24–29)^.
03	Pressure redistribution	Adopt pressure redistribution techniques, such as frequent position changes and the use of specialized support surfaces, including high-density foam mattresses, alternating mattresses, and gel or foam pillows. Record the effectiveness of these interventions in preventing complications associated with bony prominences^(19-22,24–29)^.
04	Skin Integrity Maintenance	Ensure the patient’s skin is clean and dry. Apply protective barriers to areas vulnerable to moisture (urinary or fecal incontinence) and use moisturizers to prevent dryness. Regularly monitor skin integrity and adjust care as needed^(21,22,26–29)^.
05	Pressure Ulcer Treatment	Clean and treat PU wounds according to established protocols, including the use of analgesics and anti-infective treatment when necessary. Monitor wound progression and adjust treatment as indicated^(20)^.
06	Nutritional and Water Support	Identify food preferences, track malnutrition, and monitor nutritional status. Implement protein supplementation or enteral/parenteral nutrition when indicated. Encourage fluid and food intake, adjusting consistency to facilitate intake, according to recommended nutritional protocols^(21–23,25–29)^.
07	Patient and Family Education and Involvement	Include patients and families in the care plan for PU prevention. Provide clear guidance on necessary care, warning signs, and preventive measures. Ensure they understand and are able to implement the guidelines^(19,20,22,24,28)^.
08	Health Team Training	Develop continuing education programs for healthcare staff on risk assessment, preventive interventions, and PU management. Ensure the team is up to date with best practices and protocols^(22,26)^.
09	Implementation of Standardized Instruments	Apply protocols, checklists or standardized instruments, such as SSKIN *Care Bundle, Malnutrition Universal Screening Tool* (MUST) and Safety Cross, to ensure a systematic and consistent approach to PU prevention^(23,25–28)^.
10	Technology Use	Use advanced technological systems, such as *Continuous Bedside Pressure Mapping* (CBPM), to continuously monitor the risk of PU. Integrate technological data into care practices to optimize injury prevention and response^(24)^.

To facilitate understanding of PU prevention strategies in hospitalized older adults, interventions were organized into a circular model presenting four interrelated areas, each representing fundamental aspects of the care required (Figure 2).

The “Risk Assessment and Identification” area represents the foundation of preventive interventions. It includes initial risk assessment, which is essential for identifying vulnerable patients, and ongoing reassessment, which allows monitoring the patient’s condition over time.

Next, the “Direct Practical Interventions” area encompasses specific actions, such as pressure redistribution, maintaining skin integrity and treating existing lesions. These practices aim to prevent the emergence of new lesions and minimize damage to the existing ones.

The “Additional Support and Training” area highlights the importance of providing nutritional and water support, promoting education and involvement of patients and families, and training the healthcare team. These measures increase the effectiveness of preventive interventions and strengthen collaborative care.

Finally, the “Instruments and Technology” area brings together the most advanced elements of care, including the implementation of standardized instruments and the use of technologies to improve the efficiency and accuracy of preventive interventions.

## DISCUSSION

The findings of this study allowed grouping the PU prevention strategies adopted by the nursing team in hospitalized older people into ten categories. These recommendations are aligned with international guidelines of *Prevention and Treatment of Pressure Ulcers/Injuries: Quick Reference Guide* 2019, which, although general, guide the adaptation of preventive practices to the hospital context and the older population^(30)^.

Systematic skin assessment is an essential measure for prevention. The literature recommends a holistic inspection, from head to toe, with special attention to bony prominences and areas under devices, to identify early signs of injury. This assessment should be periodic and consider support surfaces, skin integrity, mobility, comfort and activity level, to support appropriate interventions^(31)^.

Evidence indicates that predictive nursing interventions are more effective than conventional approaches in preventing PU in older people bedridden for long periods, increasing patient satisfaction and presenting high viability^(19)^. The Braden (1987) and Norton (1962) scales are widely used tools to stratify the risk of PU, by analyzing factors such as mobility, moisture, sensitivity, nutrition and friction, favoring the implementation of targeted preventive measures^(32,33)^.

Periodic reassessment and continuous monitoring enhance these results. One of the included studies showed that the use of a boot with a heel suspension device in patients over 70 years of age reduced the incidence of PU when the risk was assessed from pre-hospital care to discharge, ensuring continuity of care^(27)^.

Thus, the initial assessment, reassessment and monitoring strategies, included in the “Risk Assessment and Identification” category, are low-cost, widely applicable and highly effective for the early identification of risk factors and the timely triggering of preventive interventions.

Pressure redistribution, through changing position and using support surfaces, is an essential strategy for preventing PU. A systematic review, however, points to a lack of consensus regarding the ideal repositioning intervals, varying between 2, 3, 4 and 6 hours^(34)^. Thus, the guidelines of the *Wound Ostomy & Continence Nurses Society* recommend adjusting the frequency of repositioning to clinical conditions and individual needs, setting this care as standard^(35)^.

Support surfaces help relieve pressure on vulnerable areas and can be classified as systemic and local. The former include high-specification mattresses, reactive or alternating pressure air mattresses, and other advanced devices, widely used to protect areas such as the sacrococcygeal region, which are more susceptible to developing PU. Local surfaces, such as gel or air cushions, are adapted to anatomical specificities. Older devices such as water mattresses and pads are not recommended due to their limited effectiveness and the risks associated with improper use^(30,36)^.

Although older studies already highlighted the importance of pressure redistribution^(29)^, current guidelines reinforce updated practices, such as the 30° lateral position and the use of heel suspension devices, combined with individualized plans that balance risk, comfort and advanced technologies^(29,30)^.

Maintaining skin integrity is another central component. In environments with a high prevalence of cutaneous xerosis, incontinence-associated dermatitis, lacerations, and intertrigo, frequent inspections and maintenance of dry skin are essential measures. Evidence shows that structured care programs, including gentle cleansers and products, *leave-on*, are effective in preventing harm, especially in older people and pediatric patients^(37)^.

In one of the studies analyzed, the use of specific diagnoses and care plans for older adults contributed to prevention and improved quality of care, with interventions focused on hygiene, incontinence management, skin and nutrition monitoring, positioning and monitoring of pressure on vulnerable areas^(21)^.

Although the focus is on prevention, treatment of PU is an integral part of care. It includes proper cleaning, use of dressings and therapeutic techniques to speed healing and prevent complications. Debridement — surgical, autolytic, enzymatic, or mechanical — is essential for removing necrotic tissue, and infection control requires the use of topical or systemic antimicrobials, keeping the wound clean and covered to reduce risks and promote healing^(38)^.

A study showed that continuous nursing care reduced complications and increased satisfaction among hospitalized elderly patients, integrating prevention, treatment, and physical and psychological support, with the active participation of patients and family members^(20)^.

In the circular diagram, strategies for redistributing pressure, maintaining skin integrity, and treating PUs make up the “Direct Practical Interventions” area, which are essential for preventing new injuries and promoting the recovery of existing ones through specialized care.

Within the scope of the “Additional Support and Training” area, adequate nutrition and hydration are essential components for PU prevention and healing. The nursing team must monitor nutritional status and encourage water intake, as malnutrition and dehydration increase morbidity and mortality and compromise healing. Unplanned weight loss and low fluid intake are risk factors that require early identification and timely management^(39)^.

Current guidelines recommend a multidisciplinary approach to PUs management^(30)^. Nutritionists and dietitians adjust the diet and recommend protein supplements according to individual needs, optimizing nutritional status and promoting prevention and healing^(40)^. However, evidence regarding the effectiveness of specific nutritional compositions is still scarce^(41)^. A cross-sectional, multicenter study with patients ≥70 years old at risk of PU showed that the nutritional care provided falls short of what is recommended: nutritional screening, referral to a nutritionist, and protein supplementation are infrequent practices in routine care^(23)^.

Involving patients and family members in the care plan is another pillar of this area, including guidance on warning signs and preventive measures. This strategy, however, is rarely implemented due to the lack of adequate educational materials; when available, they are often incomplete, neglecting guidelines for caregivers and visual aids^(42)^. One of the studies analyzed reinforces the importance of integrating PU prevention into the overall care of hospitalized older individuals, highlighting the role of interdisciplinary geriatric assessment teams^(28)^.

Staff training is crucial for patient education and quality of care. Continuing education programs, whether in-person or distance learning, must be structured, supervised, and use active methodologies, such as simulation and online teaching. Evidence indicates that training lasting between 45 minutes and two hours, even in e-learning format, is effective in improving early risk identification and the implementation of preventive measures^(43,44,45)^.

In a retrospective study conducted at the Second Affiliated Hospital of Wenzhou Medical University, 103 hospitalized older individuals were assisted by newly trained nurses on the causes, prevention, and treatment of PU. Comprehensive care significantly reduced the risk of PU, improved patients’ psychological status and quality of life, and increased comfort during hospitalization^(22)^.

In the circular model, nutritional and water support strategies, associated with the education and engagement of patients and families, make up the “Additional Support and Training” area. These measures are essential for preventing PU in hospitalized older individuals, a population especially susceptible to malnutrition and dehydration, and require comprehensive, interdisciplinary, and continuous care.

In the “Instruments and Technology” area of the circular diagram, the implementation of structured protocols is a central axis for the prevention of PU. Strict adherence to well-defined protocols improves the quality of care and significantly reduces the incidence of these injuries. Specific programs enhance their effectiveness by ensuring comprehensive and continuous patient care^(46)^. In this process, nursing plays a central role in applying, monitoring and adjusting measures, adapting them to individual needs^(47)^.

The review identified essential tools for PU prevention. THE SSKIN *Care Bundle* is a comprehensive approach based on five pillars: adequate support surfaces, regular skin monitoring, moisture management, nutrition, and patient movement. The *Malnutrition Universal Screening Tool* (MUST) assesses the risk of malnutrition through factors such as Body mass index (BMI), recent weight loss, and impact of disease on nutritional status. The Safety Cross or Safety Calendar is a visual strategy used in healthcare facilities to monitor adverse events and promote a safer care environment^(26)^.

Advanced technologies have proven effective as prevention strategies. Machine learning methods for predictive models, posture recognition and image analysis contribute to early detection and continuous monitoring^(48)^. Wearable sensors aid in adherence to repositioning protocols, reducing the incidence of PU^(49)^. Other technologies, such as ultrasound, thermography, subepidermal moisture measurement, and laser Doppler spectroscopy, overcome the limitations of traditional visual inspection, enabling a more accurate and proactive approach^(50)^.

Despite these advances, technological implementation in hospitals, especially in developing countries, faces challenges: lack of infrastructure, high costs, the need for ongoing training, budgetary impact, unequal access, and dependence on imported equipment. Such factors reinforce the importance of strategies adapted to local reality, seeking accessibility and sustainability^(51)^.

One study included in this review evaluated the use of *Continuous Bedside Pressure Mapping* (CBPM) in an orthopedic rehabilitation unit in Uppsala, Sweden, with patients over 65 years of age. CBPM uses a mat with pressure sensors connected to a monitor, which indicates areas of highest pressure in red. This real-time visualization provided feedback to staff and patients, encouraging positional change and promoting self-care. After the intervention, there was an improvement in patients’ knowledge about risk factors and PU prevention, with greater engagement in preventive measures^(24)^.

In the circular model, the implementation of standardized instruments and the use of technologies make up the “Instruments and Technology” area. These strategies require more resources and involve more complex interventions than direct management actions, but they structure and qualify care practices, providing support to other areas, strengthening secondary and tertiary prevention, and contributing to reducing harm to hospitalized older people.

Despite evidence of the effectiveness of targeted interventions, the global prevalence of pressure injuries remains high. This paradox stems from structural and organizational barriers, such as a shortage of human and material resources, low adherence to guidelines, knowledge and skills gaps, a lack of a consolidated safety culture, and practical difficulties in incorporating recommendations into routine care. Even in hospitals with structured systems, a recent study shows that the implementation of guidelines is hampered by these factors, reinforcing that isolated measures are insufficient without institutional changes and consistent policies^(52)^.

A limitation of this review is the lack of formal assessment of the methodological quality of the studies included, which may affect the robustness of the conclusions. This limitation was mitigated by a comprehensive analysis of the scientific literature and by consulting recommendations from specialized bodies. Although scoping reviews do not usually perform a critical evaluation of studies, we sought to base the strategies presented on the specificities of hospitalization of older people, with recommendations more applicable to the clinical context.

This study contributes to nursing practice by identifying evidence that guides the implementation of systematized strategies for the prevention of PU. The need for continuous training of the team is emphasized, as well as the rational use of technologies for risk monitoring and evaluation of interventions, promoting greater safety and quality of care. In the hospital context, these strategies must be adapted to the available resources and infrastructure, especially in scenarios with technological and professional training limitations. Therefore, integrating these actions into institutional protocols, investing in continuing education, and using emerging technologies judiciously are essential measures to consolidate PU prevention as a safe and effective care standard.

Finally, the findings of this review are in line with the international literature, which emphasizes the need to expand nursing research on PU prevention, reinforcing the importance of evidence-based practices to adequately serve this vulnerable population^(53)^.

## CONCLUSION

The PU prevention strategies mapped in the review were organized into four areas: risk assessment and identification, direct practical interventions, additional support and training, and tools and technology. These strategies allow the nursing team to act quickly and efficiently, employing integrated interventions, optimized by professional training and the use of standardized instruments and advanced technologies.

These approaches have direct implications for care of older people. Early assessment identifies risks and enables immediate action. Practical interventions, such as pressure redistribution and skin care, prevent the development of lesions. Additional support, including nutrition, education, and staff training, enhances the quality of care. The use of standardized instruments and advanced technologies facilitates both the implementation and monitoring of preventive practices. When applied in an integrated manner, these strategies ensure quality care, reduce the risk of PU, and promote comprehensive and specific care for hospitalized older individuals.

Intervention studies exploring different strategies are recommended, with a special focus on implementing advanced technologies and rigorous protocols. These studies can strengthen the effectiveness of preventive practices and promote improvements in the quality of care provided to this vulnerable population.

## Data Availability

The entire dataset that supports the findings of this study was published within the article itself.
