# Supplementary material for: Strategies adopted by the nursing team for the prevention of pressure ulcers in hospitalized older people: a scoping review
Source: Rev Esc Enferm USP. 2025 Nov 21;59:e20240362. doi: 10.1590/1980-220X-REEUSP-2024-0362en (PMC12645846; doi:10.1590/1980-220X-REEUSP-2024-0362en)
Supplement: Translation

# Translation: Estratégias adotadas pela equipe de enfermagem para prevenção de lesões por pressão em pessoas idosas hospitalizadas: revisão de escopo

**DOI:** 10.1590/1980-220X-REEUSP-2024-0362pt

**Authors:** Maria Clara Nascimento Oliveira, Antonio Rosa de Sousa, Esteffany Vaz Pierot, Winner Gomes Machado, Francisco Gilberto Fernandes Pereira, Ana Larissa Gomes Machado

**Affiliations:** 1 Universidade Federal do Piauí, Programa de Pós-Graduação em Enfermagem, Teresina, PI, Brasil.; 2 Universidade Federal do Ceará, Programa de Pós-Graduação em Enfermagem, Fortaleza, CE, Brasil.

**Keywords:** Saúde do Idoso, Idoso, Úlcera por Pressão, Enfermagem, Cuidados de Enfermagem

## Abstract

**Objetivo:**

Mapear as estratégias descritas na literatura que podem ser adotadas pela equipe de enfermagem para a prevenção de lesões por pressão em pessoas idosas hospitalizadas.

**Método:**

Revisão de escopo estruturada conforme a metodologia do JBI, realizada em 17 de junho de 2024. A busca dos documentos foi realizada em oito bases de dados e em uma biblioteca de teses e dissertações, com auxílio do aplicativo Rayyan para a seleção dos estudos.

**Resultados:**

Foram incluídos 11 estudos na revisão. A avaliação inicial de risco, a reavaliação contínua e o monitoramento, a redistribuição da pressão, a manutenção da integridade da pele, o tratamento de lesões por pressão, o suporte nutricional e hídrico, a educação e o envolvimento de pacientes e familiares, a capacitação da equipe de saúde, além da implementação de instrumentos padronizados e do uso de tecnologia, foram identificados como as principais estratégias de prevenção.

**Conclusão:**

As estratégias de prevenção identificadas permitem à equipe de enfermagem agir de forma rápida, utilizando intervenções integradas que são otimizadas pela capacitação dos profissionais, bem como pelo uso de instrumentos padronizados e tecnologias avançadas.

## INTRODUÇÃO

A Lesão por Pressão (LP) é um dano localizado na pele ou tecidos subjacentes, geralmente sobre proeminências ósseas ou associadas a dispositivos médicos, decorrente de pressão prolongada isolada ou combinada ao cisalhamento, influenciada por fatores intrínsecos e extrínsecos que comprometem a resistência tecidual^(1)^. A prevalência global é de 12,8%, predominando os estágios I (43,5%) e II (28,0%), e os locais mais acometidos são sacro (37,3%), calcanhares (29,5%) e quadril (7,8%)^(2)^. A pressão sustentada sobre essas regiões leva à isquemia e necrose tecidual; a fricção, o cisalhamento e a umidade agravam o processo, favorecendo a ruptura da pele^(3)^.

O risco é maior em pessoas com mobilidade ou sensibilidade reduzidas, como pacientes acamados ou em cadeira de rodas, especialmente na população idosa, cuja pele apresenta alterações relacionadas ao envelhecimento^(4)^. A previsão de 2,1 bilhões de pessoas com 60 anos ou mais até 2050 evidencia a magnitude desse desafio e a necessidade de políticas e práticas preventivas eficazes^(5–7)^. No ambiente hospitalar, a prevenção de LP é uma responsabilidade compartilhada, com destaque para o papel do enfermeiro no cuidado direto. Entretanto, limitações de conhecimento e habilidades ainda comprometem a prática preventiva, reforçando a necessidade de atualização profissional^(8)^. A prática baseada em evidências é essencial para decisões clínicas seguras e eficazes, mas enfrenta barreiras como lacunas formativas e dificuldades na incorporação de recomendações^(9)^.

Apesar de campanhas e avanços tecnológicos, a incidência e o impacto das LP em pessoas idosas hospitalizadas permanecem alarmantes, com poucos avanços significativos nas últimas décadas. Esse cenário evidencia a necessidade urgente de intensificar esforços para conscientização, prevenção e tratamento dessas lesões, além de maior investimento em pesquisa e prática clínica para desenvolver estratégias mais eficazes^(10)^.

No Brasil, regulamentações como a Lei do Exercício Profissional da Enfermagem (Lei nº 7.498/1986), a Resolução COFEN nº 564/2017 e o Programa Nacional de Segurança do Paciente (PNSP), instituído pela Portaria do Ministério da Saúde nº 529/2013, estabelecem diretrizes claras para a atuação dos enfermeiros na prevenção e tratamento das LP. Essas normas reforçam a importância de práticas seguras e qualificadas para garantir a segurança e a qualidade do atendimento às pessoas idosas hospitalizadas^(11–13)^.

Apesar dessas regulamentações e da obrigatoriedade de Núcleos de Segurança do Paciente (NSP), o Brasil enfrenta desafios importantes na prevenção de LP. Entre 2014 e 2022, as LP corresponderam a 20,3% das notificações de eventos adversos no Sistema Nacional de Vigilância Sanitária (SNVS), totalizando 223.378 casos. Esse número posiciona as LP como o segundo tipo de evento adverso mais frequente no país. Nesse período, foram reportados 26.735 “never events”, dos quais 72,21% estavam relacionados a LP estágio 3 e 21,57% a LP estágio 4, com 65 óbitos diretamente atribuídos a essas lesões^(14)^.

Esses dados evidenciam que, apesar dos esforços normativos, persistem desigualdades regionais, insuficiência de recursos e lacunas na capacitação profissional. Muitos serviços hospitalares apresentam infraestrutura inadequada, incluindo falta de colchões de redistribuição de pressão e dispositivos essenciais, o que compromete as práticas preventivas. Além disso, disparidades socioeconômicas e o aumento da demanda por internações de pessoas idosas sobrecarregam o sistema de saúde, dificultando a implementação de estratégias baseadas em evidências^(14)^.

Para garantir o ineditismo desta revisão, realizou-se busca na literatura científica, abrangendo diversas bases de dados. Embora existam diretrizes globais e revisões sistemáticas sobre a prevenção de LP, essas recomendações são amplas e voltadas para equipes multiprofissionais ou instituições de longa permanência. Contudo, inexistem revisões focadas exclusivamente na atuação da enfermagem no contexto hospitalar.

Diante disso, esta revisão busca mapear as estratégias adotadas pela enfermagem e identificar as práticas preventivas mais relevantes para esse cenário. A escolha pela revisão de escopo permite uma visão abrangente, destacando não apenas as evidências consolidadas, mas também as lacunas existentes na literatura sobre a atuação da enfermagem na prevenção de LP em pessoas idosas hospitalizadas.

A relevância deste estudo reside na sistematização de estratégias preventivas para pessoas idosas hospitalizadas, um grupo particularmente vulnerável, com potencial para aprimorar práticas clínicas e garantir cuidados mais eficazes. Além disso, ao mapear os estudos realizados pela enfermagem, reforça-se o papel dessa profissão na produção de conhecimento e na implementação de intervenções preventivas. Como protagonista no cuidado contínuo, a enfermagem pode utilizar essas evidências para fomentar capacitações, influenciar políticas institucionais e fortalecer sua autonomia na prevenção de LP. Assim, esta revisão de escopo tem como objetivo mapear as estratégias descritas na literatura que podem ser adotadas pela equipe de enfermagem para a prevenção de LP em pessoas idosas hospitalizadas.

## MÉTODO

### Tipo de Pesquisa

Trata-se de uma revisão de escopo conduzida conforme a metodologia do JBI para este tipo de estudo^(15)^, seguindo as diretrizes do *Preferred Reporting Items for Systematic Reviews and Meta-Analyses 2020* (PRISMA 2020)^(16)^. O protocolo da revisão foi registrado no *Open Science Framework* (OSF) sob o Digital Object Identifier (DOI): 10.17605/OSF.IO/WRVZ5. Todas as modificações metodológicas realizadas durante o desenvolvimento do protocolo foram documentadas nesta versão.

### Pergunta de Pesquisa da Revisão

Para formular a questão de pesquisa, foi utilizada a estratégia PCC (População, Conceito e Contexto): População (pessoas idosas hospitalizadas), Conceito (estratégias de prevenção de LP) e Contexto (assistência de enfermagem a pessoas idosas hospitalizadas). Assim, a pergunta foi elaborada da seguinte forma: “Quais estratégias descritas na literatura podem ser adotadas pela equipe de enfermagem para a prevenção de LP em pessoas idosas hospitalizadas?”.

### Critérios de Elegibilidade

Os critérios de inclusão e exclusão foram estabelecidos com base no método PCC (População, Conceito e Contexto)^(15)^. A população-alvo incluiu pessoas idosas hospitalizadas, excluindo estudos que abordaram outras faixas etárias. O conceito focou em estratégias de prevenção de LP, enquanto estudos que não trataram desse tema em pessoas idosas foram excluídos. O contexto considerou a assistência de enfermagem em pessoas idosas hospitalizadas. Foram incluídas diversas fontes de evidência, sem restrição de idioma, recorte temporal ou país de origem, contemplando artigos, dissertações e teses. Excluíram-se estudos metodológicos para tradução ou validação de instrumentos, ensaios teóricos e editoriais

### Estratégia de Pesquisa

Para elaborar a estratégia de busca desta revisão de escopo, foi realizada uma pesquisa preliminar nas bases de dados PubMed^®^ e Web of Science™, na qual se utilizaram os cabeçalhos de assuntos médicos da Medline (MeSH), os descritores em Ciências da Saúde (DeCS) e o tesauro da Emtree. A leitura dos títulos e resumos dos textos encontrados nessa pesquisa preliminar permitiu a identificação de outras palavras-chave e termos sinônimos, com o objetivo de expandir os resultados da busca e obter uma estratégia mais sensível para a segunda fase de seleção dos dados.

Dessa forma, as bases de dados incluíram: PubMed^®^ (*National Library of Medicine*, NLM); Web of Science™ – Coleção Principal (*Clarivate Analytics*); Scopus^®^ (Elsevier); e Embase^®^ (Elsevier). As bases de dados Base de Dados em Enfermagem (BDENF), Literatura Latino-Americana e do Caribe de Informação em Ciências da Saúde (LILACS) e *Índice Bibliográfico Español en Ciencias de la Salud* (IBECS) foram acessadas por meio da Biblioteca Virtual em Saúde (BVS). Para contemplar estudos de literatura cinzenta, também foi utilizada a Biblioteca Digital Brasileira de Teses e Dissertações (BDTD) da Coordenação de Aperfeiçoamento de Pessoal de Nível Superior (CAPES).

Os termos relacionados ao acrônimo PCC foram adaptados para cada plataforma de dados, levando em consideração as variações e os operadores booleanos (AND e OR) para desenvolver as estratégias finais, apresentadas no Quadro 1.

**Quadro 1:** Sintaxe de construção, descritores/palavras-chaves e operadores booleanos utilizados nas bases de dados – Teresina, PI, Brasil, 2024.

Base de dados	Estratégia de busca
PubMed^®^ via NLM N = 1210	((“health of the elderly”[All Fields]) OR (“aged”[MeSH Terms]) OR (“health services for the aged”[MeSH Terms]) OR (“aged, 80 and over”[MeSH Terms]) OR (“hospitalization”[MeSH Terms])) AND ((“pressure ulcer”[MeSH Terms]) OR (“risk assessment”[MeSH Terms]))) AND ((“nursing”[MeSH Terms]) OR (“nursing, team”[MeSH Terms]) OR (“nursing care”[MeSH Terms]) OR (“nurses improving care for health system elders”[MeSH Terms]))
Web of Science™ Coleção Principal N = 1068	ALL = ((Aged* OR “Health of the Elderly” OR “Health Services for the Aged” OR “Health of the Elderly” OR “Aged, 80 and over” OR Hospitalization*) AND (Pressure Ulcer* OR “Risk Assessment”) AND (Nursing* OR Nursing, Team* OR “Nursing Care” OR “Nurses Improving Care for Health System Elders”))
Scopus^®^ N = 622	((Aged* OR “Health of the Elderly” OR “Health Services for the Aged” OR “Health of the Elderly” OR “Aged, 80 and over” OR Hospitalization*) AND (Pressure Ulcer* OR “Risk Assessment”) AND (Nursing* OR Nursing, Team* OR “Nursing Care” OR “Nurses Improving Care for Health System Elders”))
Embase^®^ N = 1688	(aged* OR ‘health services for the aged’/exp OR ‘health services for the aged’ OR ‘health of the elderly’ OR ‘aged, 80 and over’/exp OR ‘aged, 80 and over’ OR hospitalization*) AND ((‘pressure’/exp OR pressure) AND ulcer* OR ‘risk assessment’/exp OR ‘risk assessment’) AND ((nursing* OR ‘nursing,’/exp OR nursing,) AND team* OR ‘nursing care’/exp OR ‘nursing care’ OR ‘nurses improving care for health system elders’/exp OR ‘nurses improving care for health system elders’) AND [embase]/lim
BDENF, LILACS e IBECS via BVS N = 278	((“Saúde do Idoso”) OR (idos*) OR (“Serviços de Saúde para Idosos”) OR (“Saúde do Idoso”) OR (“Idoso de 80 Anos ou mais”) OR (Hospitalizaç*)) AND ((“Úlcera por Pressão”) OR (“Medição de Risco”)) AND ((“Enfermagem”) OR (“Equipe de Enfermagem”) OR (“Cuidados de Enfermagem”) OR (“Cuidado de Enfermagem ao Idoso Hospitalizado”))
BDTD da CAPES N = 50	(“Saúde do Idoso” OR idoso OR “Serviços de Saúde para Idosos” OR “Saúde do Idoso” OR “Idoso de 80 Anos ou mais” OR Hospitalização) AND (“Úlcera por Pressão” OR “Medição de Risco”) AND (“Enfermagem” OR “Equipe de Enfermagem” OR “Cuidados de Enfermagem” OR “Cuidado de Enfermagem ao Idoso Hospitalizado”)

As buscas foram realizadas em 17 de junho de 2024, utilizando acesso remoto às bases de dados por meio do portal de periódicos da Coordenação de Aperfeiçoamento de Pessoal de Nível Superior (CAPES), acessado via Comunidade Acadêmica Federada (CAFe) com o login da Universidade Federal do Piauí (UFPI).

### Seleção da Fonte de Evidências

A seleção do material relacionado à temática ocorreu com o auxílio do sistema de gerenciamento de referências *Rayyan CQRI Systems* ^(17)^. Inicialmente, os arquivos contendo a literatura encontrada em cada fonte de informação foram exportados para o sistema. Dois revisores, de forma independente, realizaram a exclusão do material duplicado. Em seguida, procederam à leitura dos resumos dos textos remanescentes, atribuindo conceitos de aceitação ou rejeição conforme os critérios de inclusão e exclusão estabelecidos. O processo foi conduzido às cegas, utilizando o recurso *blind on* disponibilizado pelo sistema, e quaisquer discrepâncias entre os revisores foram solucionadas por um terceiro revisor.

Após essa etapa, os textos selecionados foram lidos integralmente e avaliados segundo os critérios de inclusão e exclusão. Os textos aceitos foram utilizados para a extração de dados. Os resultados desse processo foram organizados em um diagrama que apresenta as fases de identificação, triagem, inclusão e seleção dos textos para a revisão.

### Extração dos Dados

A extração dos dados para inclusão nesta revisão foi realizada com base na lista de referências gerada no Rayyan, que foi exportada para uma planilha do Microsoft Excel. Posteriormente, os três revisores preencheram a planilha de forma independente, seguindo um formulário elaborado especificamente para essa etapa, com o objetivo de atender ao propósito e à questão da revisão. Durante a fase de protocolo, foi desenvolvido e testado um rascunho de tabela para o registro das principais informações. Esse rascunho foi refinado ao longo da fase de revisão e resultou na versão final, que incluiu os seguintes dados extraídos de cada fonte: fonte de informação, autor(es), título, tipo, método, país, idioma, descritores/palavras-chave e estratégias de prevenção de LP em pessoas idosas realizadas pela equipe de enfermagem.

### Apresentação dos Resultados

Os achados foram sumarizados utilizando o método de redução dos dados, por meio de leitura crítica e classificação dos resultados em categorias conceituais^(18)^. Os resultados foram apresentados em figuras e quadros. O primeiro quadro detalhou as informações descritivas dos estudos, incluindo a fonte de informação, autor(es), título, tipo, método, país, idioma e descritores/palavras-chave. O segundo quadro sintetizou as estratégias de prevenção de LP em pessoas idosas hospitalizadas que podem ser aplicadas pela equipe de enfermagem.

A Figura 1 apresenta o processo de identificação, triagem e inclusão dos artigos. A Figura 2, um diagrama circular, organiza as estratégias de prevenção de LP em pessoas idosas hospitalizadas identificadas na revisão. Diferentemente de uma hierarquia, o diagrama exibe as estratégias de forma interdependente, com intervenções posicionadas em segmentos que evidenciam sua complementaridade e relevância igualitária. Esse formato favorece uma visualização integrada das ações, ressaltando a contribuição de cada estratégia para a prevenção eficaz de LP no contexto das pessoas idosas hospitalizadas.

**Figura 1:** Fluxograma do processo de identificação, triagem e inclusão dos artigos – Teresina, PI, Brasil, 2024.

Fonte: adaptado de Page *et al.* (2021)^(16)^.

**Figura 2:** Diagrama circular das estratégias de prevenção de lesões por pressão em pessoas idosas realizadas pela equipe de enfermagem – Teresina, PI, Brasil, 2024.

A discussão foi fundamentada na literatura selecionada e em outros estudos científicos relacionados ao tema. Além disso, foram utilizados documentos de entidades que promovem a criação e disseminação de diretrizes baseadas em evidências, com o objetivo de educar e capacitar profissionais de saúde, além de desenvolver políticas voltadas para a melhoria da prevenção e tratamento de LP.

### Aspectos Éticos

Por se tratar de uma revisão de escopo sem envolvimento direto de participantes humanos, não houve necessidade de submissão ao Comitê de Ética em Pesquisa, conforme orientações do JBI. Todas as etapas foram conduzidas em conformidade com os princípios éticos aplicáveis à pesquisa, assegurando transparência, rigor metodológico e integridade científica. Foram respeitados os direitos autorais e a correta citação das fontes, garantindo a reprodutibilidade e a confiabilidade dos achados.

## RESULTADOS

As estratégias de busca identificaram um total de 4.867 publicações. Após a remoção de 412 duplicatas, restaram 4.455 estudos para a triagem dos títulos e resumos. Desses, 97 foram selecionados para leitura completa. Durante essa fase, 13 estudos não puderam ser recuperados, resultando em 84 artigos que foram lidos na íntegra. Após uma análise minuciosa, 73 artigos foram excluídos, restando 11 estudos para compor esta revisão. Todos os motivos de exclusão foram documentados visando à construção do relatório de excluídos para posterior exposição. É importante destacar que não foi possível identificar estudos adicionais por outros métodos, não adicionando mais evidências à presente revisão (Figura 1).

A partir da análise dos 11 estudos incluídos, constatou- se que a revista com o maior número de publicações sobre o tema foi a *International Wound Journal*, com duas publicações (18,18%). Além disso, os estudos foram realizados por 36 autores diferentes. Os anos com maior número de publicações foram 2021, 2019 e 2015, cada um com duas publicações (18,18%), enquanto os demais anos registraram uma publicação (9,09%) cada. Em relação à tipologia, verificou-se que todos os estudos analisados eram artigos científicos, sendo nove pesquisas de campo (81,82%). O método mais utilizado foi o quase- experimental, presente em três estudos (27,27%). Ademais, a China foi o país de origem mais representativo, com três estudos (27,27%).

No que diz respeito ao idioma, a maioria dos textos estava disponível em inglês (72,73%). Por fim, as palavras-chave destacaram estratégias de prevenção e tratamento de LP em pessoas idosas, enfatizando a importância dos cuidados de enfermagem contínuos, da terapia nutricional e do uso de tecnologia para feedback em tempo real. Essas informações estão detalhadas no Quadro 2.

**Quadro 2:** Fontes de informação selecionadas – Teresina, PI, Brasil, 2024.

Nº	Fonte e ano	Autores	Título	País/idioma	Palavras-chave	Método	Resultados
01	*Int. Wound J.* 2024	Deng et al.^(19)^	*Effects of predictive nursing interventions on pressure ulcer in elderly bedridden patients*	China/Inglês	*Bedridden; Efficacy; Elderly;* *Predictive Nursing; Pressure Ulcers*	Quase-experimental, comparando grupo controle (cuidado convencional) e grupo intervenção (enfermagem preditiva), n = 120.	A incidência de LP foi significativamente menor no grupo intervenção (10%) comparado ao controle (38.3%) (p < 0.001). Os escores da escala de Braden foram maiores no grupo intervenção (p < 0.001). Tempo de início das LP foi maior no grupo intervenção (14.57 ± 3.21 dias vs. 6.25 ± 2.36 dias, p < 0.001). Os escores de ansiedade (SAS) e depressão (SDS) foram menores no grupo intervenção após o cuidado (p < 0.001). A satisfação dos pacientes foi maior no grupo intervenção (98.3% vs. 85%, p = 0.008).
02	*Am. J. Transl. Res.* 2021	Xiao et al.^(20)^	*The preventive effect of seamless nursing care on pressure ulcer and related complications in elderly inpatients*	China/Inglês	*Seamless Nursing Care; Elderly Inpatients; Pressure Ulcer; Complications*	Ensaio clínico randomizado com 132 pessoas idosas hospitalizadas (≥65 anos), divididos em grupo controle (cuidados convencionais, n = 66) e grupo intervenção (cuidados convencionais + enfermagem contínua, n = 66).	A incidência de LP foi menor no grupo intervenção (3.03%) comparado ao controle (21.21%) (p = 0.001). A taxa de complicações foi menor no grupo intervenção (9.09% vs. 33.33%, p < 0.05). Tempo médio de internação foi reduzido no grupo intervenção (21.34 ± 4.38 vs. 26.24 ± 6.11 dias, p < 0.001). A satisfação dos pacientes foi maior no grupo intervenção (83.33% vs. 59.09%, p = 0.002).
03	*Rev. Esc. Enferm.* 2021	Morrudo Garcia et al.^(21)^	Diagnóstico de enfermagem em pessoa idosa com risco para lesão por pressão	Brasil/Português	*Idoso; Hospitalização; Lesão por Pressão; Diagnóstico de Enfermagem; Cuidados de Enfermagem*	Estudo exploratório, descritivo, transversal, quantitativo, realizado com 87 prontuários de pessoas idosas hospitalizadas em uma unidade de clínica médica de um hospital universitário no Brasil.	24 pessoas idosas (27.6%) apresentaram risco para LP. Fatores associados: ser acamado, restrição de posicionamento no leito e necessidade de auxílio para locomoção (p < 0.05). Intervenções predominantes: mobilidade, controle da pressão, supervisão da pele, nutrição e higiene.
04	*Int. J. Clin. Exp. Med.* 2020	Yu et al.^(22)^	*Analysis of the effect of comprehensive nursing on pressure ulcers risk and psychological state in severe elderly patients*	China/Inglês	*Pressure Ulcers; Elderly; Severe; Psychological State; Comprehensive Nursing*	Estudo retrospectivo comparando 103 pessoas idosas graves antes (grupo controle, 2017) e 103 após (grupo intervenção, 2018) a implementação de cuidados de enfermagem abrangentes.	A incidência de LP foi significativamente menor no grupo intervenção (11.65%) comparado ao controle (39.81%) (p < 0.05). Tempo médio de formação das LP foi menor no grupo intervenção (4.53 ± 2.81 dias vs. 10.81 ± 3.56 dias, p < 0.05). Satisfação dos pacientes foi maior no grupo intervenção (91.14% vs. 76.67%, p < 0.05).
05	*Int. Wound J.* 2019	Eglseer et al.^(23)^	*Nutritional management of older hospitalised patients with pressure injuries*	Áustria/Inglês	*Hospital; Malnutrition; Nutrition Therapy; Pressure Injury; Pressure Ulcer*	Estudo transversal multicêntrico em 33 hospitais da Áustria com 1412 pacientes hospitalizados ≥70 anos. Avaliou intervenções nutricionais, risco de LP e estado nutricional.	48% dos pacientes estavam em risco de desenvolver LP e 5% apresentavam LP. Intervenções nutricionais mais frequentes: suporte na alimentação (50.7%), triagem nutricional (39.4%). Apenas 28.2% dos pacientes com LP foram encaminhados a um nutricionista.
06	*J. Wound Ostomy Continence Nurs.* 2019	Hultin et al.^(24)^	*Information and communication technology can increase patient participation in pressure injury prevention: a qualitative study in older orthopedic patients*	Suécia/Inglês	*Information and Communication Technology; Older Patients; Patient Participation; Pressure Injuries; Pressure Injury Prevention; Real-Time Feedback*	Estudo qualitativo em unidade de reabilitação ortopédica na Suécia com 31 pacientes ≥65 anos. Dados coletados por entrevistas semiestruturadas analisadas por análise de conteúdo.	A maioria dos pacientes (n = 21) conseguiu utilizar o *Continuous Bedside Pressure Mapping* (CBPM). Os participantes relataram maior conhecimento sobre prevenção de lesões por pressão (LP) e passaram a se reposicionar com mais frequência. Os principais benefícios foram aumento da independência no autocuidado e participação ativa na prevenção de LP. O CBPM forneceu feedback visual em tempo real, permitindo ajustes imediatos na posição dos pacientes para reduzir a pressão sobre áreas vulneráveis. A tecnologia foi considerada útil na prevenção de LP e pode ser integrada aos cuidados clínicos como um recurso padrão.
07	*Appl. Nurs. Res.* 2016	Bååth et al.^(25)^	*Prevention of heel pressure ulcers among older patients – from ambulance care to hospital discharge: A multi-centre randomized controlled trial*	Suécia/Inglês	*Heel Pressure Ulcer; Prevention; Ambulance Care; Emergency Department; Acute Care Delivery Chain; RCT*	Ensaio clínico randomizado multicêntrico com 183 pessoas idosas (≥70 anos) transportadas por ambulância. Grupo intervenção utilizou dispositivo de suspensão do calcanhar.	A incidência de LP no calcanhar foi significativamente menor no grupo intervenção (14.6%) comparado ao controle (30%) (p = 0.017). Menos dor associada às lesões foi relatada no grupo intervenção. O dispositivo de suspensão do calcanhar reduziu o tempo de contato direto da pele com superfícies de apoio, minimizando a formação de LP. Os resultados sugerem que o uso de dispositivos tecnológicos, como suportes de redistribuição de pressão, deve ser incorporado rotineiramente na assistência pré-hospitalar.
08	*Nurs. Stand.* 2015	Barry e Nugent^(26)^	*Pressure ulcer prevention in frail older people*	Irlanda/Inglês	*Education; Frail Older People; Incidence; Older People; Pressure Ulcers;* *Pressure Ulcer Prevention; Prevalence; Wound Care; Wound Management*	Revisão narrativa sobre prevenção de LP em pessoas idosas frágeis, abordando programas educacionais, liderança clínica e trabalho multidisciplinar.	Programas educacionais estruturados reduziram em 73% a incidência de LP em pessoas idosas frágeis. A abordagem SSKIN (Superfície, Inspeção da Pele, Movimentação, Incontinência, Nutrição) demonstrou eficácia na prevenção e padronização dos cuidados. O estudo reforça a necessidade do uso de tecnologias de monitoramento de pressão e superfícies especializadas para otimizar a prevenção de LP.
09	*Index Enferm.* 2015	Sousa et al.^(27)^	*Concepciones teóricas de Neuman asociadas con la prevención de las úlceras por presión: Un estudio de caso*	Brasil/Espanhol	*Úlcera por Presión; Teoría de Enfermería; Cuidado de Enfermería*	Relato de caso baseado na Teoria de Sistemas de Betty Neuman para prevenção de LP. Caso de uma idosa internada por 64 dias na Unidade de Terapia Intensiva (UTI).	A paciente não desenvolveu LP durante a internação, sugerindo eficácia das estratégias preventivas baseadas na Teoria de Neuman.
10	*Crit. Care Nurs. Clin. North Am.* 2007	Stotts e Wu^(28)^	*Hospital recovery is facilitated by prevention of pressure ulcers in older adults*	EUA/Inglês	Sem palavras-chave	Revisão narrativa sobre prevenção de LP em pessoas idosas hospitalizadas e impacto na recuperação.	A incidência de LP em pessoas idosas hospitalizadas varia entre 0.4% e 38%. A prevenção está associada a menor tempo de internação e melhores desfechos funcionais.
11	*Geriatr. Nurs.* 1997	Maklebust^(29)^	*Pressure ulcers: decreasing the risk for older adults*	EUA/Inglês	Sem palavras-chave	Revisão narrativa sobre fatores de risco e estratégias de prevenção de LP em pessoas idosas.	Os principais fatores de risco incluem imobilidade, umidade excessiva, fricção, cisalhamento e desnutrição. Medidas preventivas incluem reposicionamento, superfícies de suporte e nutrição adequada.

A análise das estratégias para prevenção da LP em pessoas idosas hospitalizadas revelou 10 abordagens importantes que podem ser realizadas pela equipe de enfermagem: avaliação inicial de risco; reavaliação contínua e monitoramento; redistribuição da pressão; manutenção da integridade da pele; tratamento de LP, suporte nutricional e hídrico; educação e envolvimento de pacientes e familiares; capacitação da equipe de saúde, implementação de instrumentos padronizados e uso de tecnologia (Quadro 3).

**Quadro 3:** Estratégias de prevenção de lesões por pressão em pessoas idosas que podem ser realizadas pela equipe de enfermagem – Teresina, PI, Brasil, 2024.

Nº	Estratégia	Descrição
01	Avaliação Inicial de Risco	Realizar avaliação inicial com escalas de risco, como Braden e Norton, para identificar pacientes com risco elevado de LP. Documentar e atualizar regularmente a avaliação para refletir mudanças no estado do paciente^(19,20,25–28)^.
02	Reavaliação Contínua e Monitoramento	Inspecionar diariamente a pele para identificar sinais precoces de LP, como alterações na coloração ou integridade. Documentar os resultados, implementar intervenções imediatas quando necessário e ajustar o plano de cuidados com base nas observações^(20–22,24–29)^.
03	Redistribuição da pressão	Adotar técnicas de redistribuição da pressão, como mudanças frequentes de decúbito e o uso de superfícies de suporte especializadas, incluindo colchões de espuma de alta densidade, colchões alternantes e almofadas de gel ou espuma. Registrar a eficácia dessas intervenções para prevenir complicações associadas a proeminências ósseas^(19–22,24–29)^.
04	Manutenção da Integridade da Pele	Garantir que a pele do paciente esteja limpa e seca. Aplicar barreiras protetoras em áreas vulneráveis à umidade (incontinência urinária ou fecal) e utilizar hidratantes para prevenir o ressecamento. Monitorar regularmente a integridade da pele e ajustar os cuidados conforme necessário^(21,22,26–29)^.
05	Tratamento de Lesões por Pressão	Limpar e tratar feridas de LP de acordo com protocolos estabelecidos, incluindo o uso de analgésicos e tratamento anti-infeccioso quando necessário. Monitorar a evolução das feridas e ajustar o tratamento conforme indicado^(20)^.
06	Suporte Nutricional e Hídrico	Identificar preferências alimentares, rastrear desnutrição e monitorar o estado nutricional. Implementar suplementação proteica ou nutrição enteral/parenteral quando indicado. Incentivar a ingestão de líquidos e alimentos, ajustando a consistência para facilitar a ingestão, conforme os protocolos nutricionais recomendados^(21–23,25–29)^.
07	Educação e Envolvimento de Pacientes e Familiares	Incluir pacientes e familiares no plano de cuidados para a prevenção de LP. Fornecer orientações claras sobre cuidados necessários, sinais de alerta e medidas preventivas. Certificar-se de que compreendam e sejam capazes de implementar as orientações^(19,20,22,24,28)^.
08	Capacitação da Equipe de Saúde	Desenvolver programas de educação contínua para a equipe de saúde sobre avaliação de risco, intervenções preventivas e manejo de LP. Assegurar que a equipe esteja atualizada com as melhores práticas e protocolos^(22,26)^.
09	Implementação de Instrumentos Padronizados	Aplicar protocolos, checklists ou instrumentos padronizados, como o SSKIN *Care Bundle, Malnutrition Universal Screening Tool* (MUST) e Cruz de Segurança, para garantir abordagem sistemática e consistente na prevenção de LP^(23,25–28)^.
10	Uso de Tecnologia	Utilizar sistemas tecnológicos avançados, como *Continuous Bedside Pressure Mapping* (CBPM), para monitorar continuamente o risco de LP. Integrar dados tecnológicos às práticas de cuidado para otimizar a prevenção e resposta às lesões^(24)^.

Para facilitar a compreensão das estratégias de prevenção de LP em pessoas idosas hospitalizadas, as intervenções foram organizadas em um modelo circular que apresenta quatro áreas inter-relacionadas, cada uma representando aspectos fundamentais dos cuidados necessários (Figura 2).

A área “Avaliação e Identificação de Risco” representa o alicerce das intervenções preventivas. Ela inclui a avaliação inicial do risco, essencial para identificar pacientes vulneráveis, e a reavaliação contínua, que permite monitorar o estado do paciente ao longo do tempo.

Na sequência, a área “Intervenções Práticas Diretas” engloba ações específicas, como a redistribuição da pressão, a manutenção da integridade da pele e o tratamento de lesões já estabelecidas. Essas práticas visam prevenir o surgimento de novas lesões e minimizar os danos de lesões existentes.

A área “Suporte Adicional e Capacitação” destaca a importância de oferecer suporte nutricional e hídrico, promover a educação e o envolvimento de pacientes e familiares, além de capacitar a equipe de saúde. Essas medidas ampliam a eficácia das intervenções preventivas e fortalecem o cuidado colaborativo.

Por fim, a área “Instrumentos e Tecnologia” reúne os elementos mais avançados do cuidado, incluindo a implementação de instrumentos padronizados e a utilização de tecnologias para melhorar a eficiência e a precisão das intervenções preventivas.

## DISCUSSÃO

Os achados deste estudo permitiram agrupar as estratégias de prevenção de LP adotadas pela equipe de enfermagem em pessoas idosas hospitalizadas em dez categorias. Essas recomendações estão alinhadas às diretrizes internacionais do *Prevention and Treatment of Pressure Ulcers/Injuries: Quick Reference Guide* 2019, que, embora gerais, orientam a adaptação das práticas preventivas para o contexto hospitalar e para a população idosa^(30)^.

A avaliação sistemática da pele é uma medida essencial para a prevenção. A literatura recomenda uma inspeção holística, da cabeça aos pés, com atenção especial às proeminências ósseas e às áreas sob dispositivos, visando identificar precocemente sinais de lesão. Essa avaliação deve ser periódica e considerar superfícies de apoio, integridade da pele, mobilidade, conforto e nível de atividade, de modo a subsidiar intervenções adequadas^(31)^.

Evidências indicam que intervenções preditivas de enfermagem são mais eficazes que abordagens convencionais na prevenção de LP em idosos acamados por longos períodos, aumentando a satisfação dos pacientes e apresentando alta viabilidade^(19)^. As escalas de Braden (1987) e Norton (1962) são ferramentas amplamente utilizadas para estratificar o risco de LP, ao analisarem fatores como mobilidade, umidade, sensibilidade, nutrição e fricção, favorecendo a implementação de medidas preventivas direcionadas^(32,33)^.

A reavaliação periódica e o monitoramento contínuo potencializam esses resultados. Um dos estudos incluídos mostrou que a utilização de bota com dispositivo de suspensão de calcanhar em pacientes acima de 70 anos reduziu a incidência de LP quando o risco foi avaliado desde o atendimento pré-hospitalar até a alta, garantindo a continuidade do cuidado^(27)^.

Assim, as estratégias de avaliação inicial, reavaliação e monitoramento, compreendidas na categoria “Avaliação e Identificação de Risco”, apresentam baixo custo, aplicabilidade ampla e elevada eficácia para a identificação precoce de fatores de risco e para o desencadeamento oportuno de intervenções preventivas.

A redistribuição da pressão, por meio da mudança de decúbito e do uso de superfícies de suporte, é uma estratégia essencial para a prevenção de LP. Uma revisão sistemática, contudo, aponta ausência de consenso quanto aos intervalos ideais de reposicionamento, variando entre 2, 3, 4 e 6 horas^(34)^. Assim, as diretrizes da *Wound Ostomy & Continence Nurses Society* recomendam ajustar a frequência do reposicionamento às condições clínicas e necessidades individuais, definindo esse cuidado como padrão^(35)^.

As superfícies de suporte contribuem para aliviar a pressão em áreas vulneráveis e podem ser classificadas em sistêmicas e locais. As primeiras incluem colchões de alta especificação, colchões de ar reativos ou de pressão alternada e outros dispositivos avançados, amplamente utilizados para proteger regiões como a sacrococcígea, mais suscetível ao desenvolvimento de LP. As superfícies locais, como almofadas de gel ou de ar, são adaptadas às especificidades anatômicas. Dispositivos antigos, como colchões e almofadas de água, não são recomendados devido à eficácia limitada e aos riscos associados ao uso inadequado^(30,36)^.

Embora estudos mais antigos já destacassem a importância da redistribuição da pressão^(29)^, diretrizes atuais reforçam práticas atualizadas, como a posição lateral a 30° e o uso de dispositivos suspensores para os calcanhares, aliadas a planos individualizados que conciliem risco, conforto e tecnologias avançadas^(29,30)^.

A manutenção da integridade da pele é outro componente central. Em ambientes de alta prevalência de xerose cutânea, dermatite associada à incontinência, lacerações e intertrigo, inspeções frequentes e manutenção da pele seca são medidas essenciais. Evidências demonstram que programas estruturados de cuidados, incluindo limpadores suaves e produtos *leave-on*, são eficazes para prevenir danos, especialmente em pessoas idosas e pacientes pediátricos^(37)^.

Em um dos estudos analisados, o uso de diagnósticos e planos de cuidados específicos para pessoas idosas contribuiu para a prevenção e para a melhoria da qualidade do cuidado, com intervenções voltadas a higiene, manejo da incontinência, monitoramento da pele e nutrição, posicionamento e supervisão da pressão sobre áreas vulneráveis^(21)^.

Embora o foco seja a prevenção, o tratamento das LP é parte integrante da assistência. Inclui limpeza adequada, uso de curativos e técnicas terapêuticas para acelerar a cicatrização e prevenir complicações. O desbridamento — cirúrgico, autolítico, enzimático ou mecânico — é essencial na remoção de tecido necrótico, e o controle da infecção requer o uso de antimicrobianos tópicos ou sistêmicos, mantendo a lesão limpa e coberta para reduzir riscos e favorecer a cicatrização^(38)^.

Um estudo mostrou que o cuidado de enfermagem contínuo reduziu complicações e aumentou a satisfação dos pacientes idosos hospitalizados, integrando prevenção, tratamento e apoio físico e psicológico, com participação ativa de pacientes e familiares^(20)^.

No diagrama circular, as estratégias de redistribuição da pressão, manutenção da integridade da pele e tratamento das LP compõem a área “Intervenções Práticas Diretas”, fundamentais para prevenir novas lesões e favorecer a recuperação das existentes por meio de cuidados especializados.

No âmbito da área “Suporte Adicional e Capacitação”, a nutrição e a hidratação adequadas são componentes essenciais para a prevenção e cicatrização de LP. A equipe de enfermagem deve monitorar o estado nutricional e incentivar a ingestão hídrica, uma vez que desnutrição e desidratação aumentam a morbimortalidade e comprometem a cicatrização. A perda de peso não planejada e a baixa ingestão de líquidos são fatores de risco que exigem identificação precoce e manejo oportuno^(39)^.

As diretrizes atuais recomendam abordagem multidisciplinar para o manejo das LP^(30)^. Nutricionistas e dietistas ajustam a dieta e indicam suplementos proteicos de acordo com as necessidades individuais, otimizando o estado nutricional e favorecendo a prevenção e a cicatrização^(40)^. Contudo, ainda são escassas as evidências quanto à eficácia de composições nutricionais específicas^(41)^. Um estudo transversal e multicêntrico com pacientes ≥70 anos em risco de LP evidenciou que o cuidado nutricional oferecido está aquém do recomendado: triagem nutricional, encaminhamento ao nutricionista e suplementação proteica são práticas pouco frequentes na rotina assistencial^(23)^.

O envolvimento de pacientes e familiares no plano de cuidados é outro pilar dessa área, abrangendo orientações sobre sinais de alerta e medidas preventivas. Essa estratégia, porém, é pouco implementada devido à ausência de materiais educativos adequados; quando disponíveis, costumam ser incompletos, negligenciando diretrizes para cuidadores e recursos visuais^(42)^. Um dos estudos analisados reforça a importância de integrar a prevenção de LP ao cuidado global da pessoa idosa hospitalizada, destacando a atuação de equipes interdisciplinares de avaliação geriátrica^(28)^.

A capacitação da equipe é decisiva para a educação do paciente e para a qualidade assistencial. Programas de educação continuada, presenciais ou a distância, devem ser estruturados, supervisionados e utilizar metodologias ativas, como simulação e ensino online. Evidências indicam que treinamentos entre 45 minutos e duas horas, mesmo em formato e-learning, são eficazes para aprimorar a identificação precoce de riscos e a implementação de medidas preventivas^(43–45)^.

Em um estudo retrospectivo realizado no Second Affiliated Hospital of Wenzhou Medical University, 103 idosos hospitalizados foram assistidos por enfermeiros recém-treinados sobre causas, prevenção e tratamento de LP. O cuidado integral reduziu significativamente o risco de LP, melhorou o estado psicológico e a qualidade de vida dos pacientes e aumentou o conforto durante a hospitalização^(22)^.

No modelo circular, as estratégias de suporte nutricional e hídrico, associadas à educação e ao engajamento de pacientes e familiares, compõem a área “Suporte Adicional e Capacitação”. Essas medidas são fundamentais para a prevenção de LP em idosos hospitalizados, população especialmente suscetível à desnutrição e à desidratação, e exigem um cuidado integral, interdisciplinar e contínuo.

Na área “Instrumentos e Tecnologia” do diagrama circular, a implementação de protocolos estruturados é um eixo central para a prevenção de LP. A adesão rigorosa a protocolos bem definidos melhora a qualidade da assistência e reduz significativamente a incidência dessas lesões. Programas específicos potencializam sua eficácia ao garantir atenção integral e contínua ao paciente^(46)^. Nesse processo, a enfermagem desempenha papel central na aplicação, monitoramento e ajuste das medidas, adaptando-as às necessidades individuais^(47)^.

A revisão identificou ferramentas essenciais para a prevenção de LP. O SSKIN *Care Bundle* é uma abordagem abrangente baseada em cinco pilares: superfícies de suporte adequadas, supervisão regular da pele, manejo da umidade, nutrição e movimentação do paciente. O *Malnutrition Universal Screening Tool* (MUST) avalia o risco de desnutrição por meio de fatores como o Índice de massa corporal (IMC), perda de peso recente e impacto da doença no estado nutricional. Já a Cruz de Segurança ou Calendário de Segurança é uma estratégia visual utilizada em unidades de saúde para monitorar eventos adversos e promover um ambiente de cuidado mais seguro^(26)^.

Tecnologias avançadas têm se mostrado eficazes como estratégias de prevenção. Métodos de aprendizado de máquina para modelos preditivos, reconhecimento de postura e análise de imagens contribuem para detecção precoce e monitoramento contínuo^(48)^. Sensores vestíveis auxiliam na adesão aos protocolos de reposicionamento, reduzindo a incidência de LP^(49)^. Outras tecnologias, como ultrassom, termografia, medição de umidade subepidérmica e espectroscopia a laser Doppler, superam as limitações da inspeção visual tradicional, possibilitando uma abordagem mais precisa e proativa^(50)^.

Apesar desses avanços, a implementação tecnológica em hospitais, especialmente em países em desenvolvimento, enfrenta desafios: falta de infraestrutura, custos elevados, necessidade de capacitação contínua, impacto orçamentário, desigualdade no acesso e dependência de equipamentos importados. Tais fatores reforçam a importância de estratégias adaptadas à realidade local, buscando acessibilidade e sustentabilidade^(51)^.

Um estudo incluído nesta revisão avaliou o uso do *Continuous Bedside Pressure Mapping* (CBPM) em uma unidade de reabilitação ortopédica em Uppsala, Suécia, com pacientes acima de 65 anos. O CBPM utiliza um tapete com sensores de pressão conectados a um monitor, que indica em vermelho as áreas de maior pressão. Essa visualização em tempo real forneceu feedback à equipe e aos pacientes, incentivando a mudança de posição e promovendo autocuidado. Após a intervenção, houve melhora no conhecimento dos pacientes sobre fatores de risco e prevenção de LP, com maior engajamento nas medidas preventivas^(24)^.

No modelo circular, a implementação de instrumentos padronizados e o uso de tecnologias compõem a área “Instrumentos e Tecnologia”. Essas estratégias demandam mais recursos e envolvem intervenções mais complexas do que as ações diretas de manejo, porém estruturam e qualificam as práticas assistenciais, oferecendo suporte às demais áreas, fortalecendo a prevenção secundária e terciária e contribuindo para reduzir os danos em pessoas idosas hospitalizadas.

Apesar das evidências de eficácia das intervenções pontuais, a prevalência global das lesões por pressão permanece elevada. Esse paradoxo decorre de barreiras estruturais e organizacionais, como a escassez de recursos humanos e materiais, baixa adesão às diretrizes, lacunas de conhecimento e habilidades, ausência de cultura de segurança consolidada e dificuldades práticas para incorporar recomendações na rotina assistencial. Mesmo em hospitais com sistemas estruturados, um estudo recente demonstra que a implementação das diretrizes é prejudicada por esses fatores, reforçando que medidas isoladas são insuficientes sem mudanças institucionais e políticas consistentes^(52)^.

Uma limitação desta revisão é a ausência de avaliação formal da qualidade metodológica dos estudos incluídos, o que pode afetar a robustez das conclusões. Essa limitação foi atenuada pela análise abrangente da literatura científica e pela consulta a recomendações de órgãos especializados. Embora revisões de escopo usualmente não realizem avaliação crítica dos estudos, buscou-se embasar as estratégias apresentadas considerando as especificidades da hospitalização de pessoas idosas, com recomendações mais aplicáveis ao contexto clínico.

Este estudo contribui para a prática de enfermagem ao identificar evidências que orientam a implementação de estratégias sistematizadas para a prevenção de LP. Ressalta-se a necessidade de capacitação contínua da equipe, bem como do uso racional de tecnologias para monitoramento de risco e avaliação de intervenções, promovendo maior segurança e qualidade assistencial. No contexto hospitalar, essas estratégias devem ser adaptadas aos recursos e à infraestrutura disponíveis, especialmente em cenários com limitações tecnológicas e de capacitação profissional. Assim, integrar essas ações aos protocolos institucionais, investir em educação continuada e utilizar tecnologias emergentes de forma criteriosa são medidas essenciais para consolidar a prevenção de LP como padrão assistencial seguro e efetivo.

Por fim, os achados desta revisão estão alinhados à literatura internacional, que enfatiza a necessidade de ampliar a pesquisa em enfermagem sobre prevenção de LP, reforçando a importância de práticas fundamentadas em evidências para atender adequadamente essa população vulnerável^(53)^.

## CONCLUSÃO

As estratégias de prevenção de LP mapeadas na revisão foram organizadas em quatro áreas: avaliação e identificação de risco, intervenções práticas diretas, suporte adicional e capacitação, e instrumentos e tecnologia. Essas estratégias permitem à equipe de enfermagem atuar de forma rápida e eficiente, empregando intervenções integradas, otimizadas pela capacitação profissional e pelo uso de instrumentos padronizados e tecnologias avançadas.

Essas abordagens apresentam implicações diretas na assistência à pessoa idosa. A avaliação precoce identifica riscos e possibilita uma ação imediata. As intervenções práticas, como a redistribuição da pressão e os cuidados com a pele, previnem o desenvolvimento de lesões. O suporte adicional, incluindo nutrição, educação e treinamento da equipe, aprimora a qualidade do cuidado. O uso de instrumentos padronizados e tecnologias avançadas facilita tanto a aplicação quanto o monitoramento das práticas preventivas. Quando aplicadas de forma integrada, essas estratégias garantem um cuidado de qualidade, reduzem o risco de LP e promovem uma assistência integral e específica para pessoas idosas hospitalizadas.

Recomenda-se a realização de estudos de intervenção que explorem as diferentes estratégias, com foco especial na implementação de tecnologias avançadas e protocolos rigorosos. Esses estudos podem fortalecer a eficácia das práticas preventivas e promover melhorias na qualidade do cuidado oferecido a essa população vulnerável.

## Data Availability

Todo o conjunto de dados que sustenta os resultados deste estudo foi publicado no próprio artigo.

# Translation: Estrategias adoptadas por el equipo de enfermería para la prevención de lesiones por presión en personas mayores hospitalizadas: revisión de alcance

**DOI:** 10.1590/1980-220X-REEUSP-2024-0362es

**Authors:** Maria Clara Nascimento Oliveira, Antonio Rosa de Sousa, Esteffany Vaz Pierot, Winner Gomes Machado, Francisco Gilberto Fernandes Pereira, Ana Larissa Gomes Machado

**Affiliations:** 1 Universidade Federal do Piauí, Programa de Pós-Graduação em Enfermagem, Teresina, PI, Brasil.; 2 Universidade Federal do Ceará, Programa de Pós-Graduação em Enfermagem, Fortaleza, CE, Brasil.

**Keywords:** Salud del Anciano, Anciano, Úlcera por Presión, Enfermería, Atención de Enfermería

## Abstract

**Objetivo:**

Mapear las estrategias descritas en la literatura que pueden ser adoptadas por el equipo de enfermería para prevenir lesiones por presión en ancianos hospitalizados.

**Método:**

Revisión de alcance estructurada según la metodología JBI, realizada el 17 de junio de 2024. La búsqueda de documentos se realizó en ocho bases de datos y en una biblioteca de tesis y disertaciones, con ayuda de la aplicación Rayyan para la selección de estudios.

**Resultados:**

Se incluyeron once estudios en la revisión. La evaluación inicial del riesgo, la reevaluación continua y el seguimiento, la redistribución de la presión, el mantenimiento de la integridad de la piel, el tratamiento de las lesiones por presión, el soporte nutricional y de líquidos, la educación y la participación de los pacientes y las familias, la capacitación del equipo de atención médica, además de la implementación de instrumentos estandarizados y el uso de tecnología, se identificaron como las principales estrategias de prevención.

**Conclusión:**

Las estrategias de prevención identificadas permiten al equipo de enfermería actuar con rapidez, utilizando intervenciones integradas y optimizadas por la formación profesional, así como el uso de instrumentos estandarizados y tecnologías avanzadas.

## INTRODUCCIÓN

La lesión por presión (LP) es un daño localizado en la piel o los tejidos subyacentes, generalmente sobre prominencias óseas o asociado a dispositivos médicos, que se produce por una presión prolongada aislada o combinada con cizallamiento, influenciada por factores intrínsecos y extrínsecos que comprometen la resistencia tisular^(1)^. La prevalencia global es del 12,8%, predominando los estadios I (43,5%) y II (28,0%), y las zonas más afectadas son el sacro (37,3%), los talones (29,5%) y la cadera (7,8%)^(2)^. La presión sostenida sobre estas regiones provoca isquemia y necrosis tisular; la fricción, el cizallamiento y la humedad agravan el proceso, favoreciendo la ruptura de la piel^(3)^.

El riesgo es mayor en personas con movilidad o sensibilidad reducidas, como pacientes encamados o en silla de ruedas, especialmente en la población de edad avanzada, cuya piel presenta alteraciones relacionadas con el envejecimiento^(4)^. La previsión de 2100 millones de personas de 60 años o más para 2050 pone de manifiesto la magnitud de este reto y la necesidad de políticas y prácticas preventivas eficaces^(5–7)^. En el entorno hospitalario, la prevención de las úlceras por presión es una responsabilidad compartida, destacando el papel del enfermero en la atención directa. Sin embargo, las limitaciones de conocimientos y habilidades aún comprometen la práctica preventiva, lo que refuerza la necesidad de actualización profesional^(8)^. La práctica basada en la evidencia es esencial para tomar decisiones clínicas seguras y eficaces, pero se enfrenta a obstáculos como las lagunas formativas y las dificultades para incorporar las recomendaciones^(9)^.

A pesar de las campañas y los avances tecnológicos, la incidencia y el impacto de las úlceras por presión en las personas mayores hospitalizadas siguen siendo alarmantes, con pocos avances significativos en las últimas décadas. Este escenario pone de manifiesto la urgente necesidad de intensificar los esfuerzos de sensibilización, prevención y tratamiento de estas lesiones, así como de aumentar la inversión en investigación y práctica clínica para desarrollar estrategias más eficaces^(10)^.

En Brasil, normativas como la Ley del Ejercicio Profesional de la Enfermería (Ley n.º 7.498/1986), la Resolución COFEN n.º 564/2017 y el Programa Nacional de Seguridad del Paciente (PNSP), instituido por la Orden del Ministerio de Salud n.º 529/2013, establecen directrices claras para la actuación de los enfermeros en la prevención y el tratamiento de las LP. Estas normas refuerzan la importancia de prácticas seguras y cualificadas para garantizar la seguridad y la calidad de la atención a las personas mayores hospitalizadas^(11–13)^.

A pesar de estas regulaciones y de la obligatoriedad de los Núcleos de Seguridad del Paciente (NSP), Brasil enfrenta importantes desafíos en la prevención de LP. Entre 2014 y 2022, las LP representaron el 20,3% de las notificaciones de eventos adversos en el Sistema Nacional de Vigilancia Sanitaria (SNVS), con un total de 223 378 casos. Esta cifra sitúa a las LP como el segundo tipo de evento adverso más frecuente en el país. En este periodo, se notificaron 26 735 “never events”, de los cuales el 72,21% estaban relacionados con LP de estadio 3 y el 21,57% con LP de estadio 4, con 65 muertes directamente atribuidas a estas lesiones^(14)^.

Estos datos evidencian que, a pesar de los esfuerzos normativos, persisten las desigualdades regionales, la insuficiencia de recursos y las deficiencias en la capacitación profesional. Muchos servicios hospitalarios presentan una infraestructura inadecuada, incluida la falta de colchones de redistribución de presión y dispositivos esenciales, lo que compromete las prácticas preventivas. Además, las disparidades socioeconómicas y el aumento de la demanda de hospitalizaciones de personas mayores sobrecargan el sistema de salud, dificultando la implementación de estrategias basadas en la evidencia^(14)^.

Para garantizar la originalidad de esta revisión, se realizó una búsqueda en la literatura científica, abarcando varias bases de datos. Aunque existen directrices globales y revisiones sistemáticas sobre la prevención de las úlceras por presión, estas recomendaciones son amplias y están dirigidas a equipos multidisciplinarios o instituciones de larga estancia. Sin embargo, no existen revisiones centradas exclusivamente en la actuación de la enfermería en el contexto hospitalario.

Ante esto, esta revisión busca mapear las estrategias adoptadas por la enfermería e identificar las prácticas preventivas más relevantes para este escenario. La elección de la revisión de alcance permite una visión amplia, destacando no solo las evidencias consolidadas, sino también las lagunas existentes en la literatura sobre la actuación de la enfermería en la prevención de las úlceras por presión en personas mayores hospitalizadas.

La relevancia de este estudio radica en la sistematización de estrategias preventivas para personas mayores hospitalizadas, un grupo particularmente vulnerable, con potencial para mejorar las prácticas clínicas y garantizar una atención más eficaz. Además, al mapear los estudios realizados por la enfermería, se refuerza el papel de esta profesión en la producción de conocimiento y en la implementación de intervenciones preventivas. Como protagonista en la atención continua, la enfermería puede utilizar estas evidencias para fomentar la capacitación, influir en las políticas institucionales y fortalecer su autonomía en la prevención de las úlceras por presión. Así, esta revisión de alcance tiene como objetivo mapear las estrategias descritas en la literatura que pueden ser adoptadas por el equipo de enfermería para la prevención de las úlceras por presión en personas mayores hospitalizadas.

## MÉTODO

### Tipo de Investigación

Se trata de una revisión de alcance realizada según la metodología del JBI para este tipo de estudio^(15)^, siguiendo las directrices de los*Preferred Reporting Items for Systematic Reviews and Meta-Analyses 2020*(PRISMA 2020)^(16)^. El protocolo de la revisión se registró en el Open Science Framework (OSF) con el Identificador de Objeto Digital (DOI): 10.17605/OSF.IO/WRVZ5. Todas las modificaciones metodológicas realizadas durante el desarrollo del protocolo se documentaron en esta versión.

### Pregunta de Investigación de la Revisión

Para formular la pregunta de investigación, se utilizó la estrategia PCC (Población, Concepto y Contexto): Población (personas mayores hospitalizadas), Concepto (estrategias de prevención de LP) y Contexto (atención de enfermería a personas mayores hospitalizadas). Así, la pregunta se formuló de la siguiente manera: “¿Qué estrategias descritas en la literatura pueden adoptar los equipos de enfermería para la prevención de las úlceras por presión en personas mayores hospitalizadas?”.

### Criterios de Elegibilidad

Los criterios de inclusión y exclusión se establecieron basándose en el método PCC (Población, Concepto y Contexto)^(15)^. La población objetivo incluyó a personas mayores hospitalizadas, excluyendo los estudios que abordaban otros grupos de edad. El concepto se centró en las estrategias de prevención de las úlceras por presión, mientras que se excluyeron los estudios que no trataban este tema en personas mayores. El contexto consideró la asistencia de enfermería a personas mayores hospitalizadas. Se incluyeron diversas fuentes de evidencia, sin restricción de idioma, periodo de tiempo o país de origen, incluyendo artículos, disertaciones y tesis. Se excluyeron los estudios metodológicos para la traducción o validación de instrumentos, ensayos teóricos y editoriales.

### Estrategia de Búsqueda

Para elaborar la estrategia de búsqueda de esta revisión de alcance, se realizó una investigación preliminar en las bases de datos PubMed^®^ y Web of Science™, en la que se utilizaron los encabezados de temas médicos de Medline (MeSH), los descriptores en Ciencias de la Salud (DeCS) y el tesauro de Emtree. La lectura de los títulos y resúmenes de los textos encontrados en esta investigación preliminar permitió identificar otras palabras clave y términos sinónimos, con el objetivo de ampliar los resultados de la búsqueda y obtener una estrategia más sensible para la segunda fase de selección de datos.

De este modo, las bases de datos incluyeron: PubMed^®^ (Biblioteca Nacional de Medicina, NLM); Web of Science™ – Colección Principal (Clarivate Analytics); Scopus^®^ (Elsevier); y Embase^®^ (Elsevier). Se accedió a las bases de datos Base de Datos en Enfermería (BDENF), Literatura Latinoamericana y del Caribe en Ciencias de la Salud (LILACS) e Índice Bibliográfico Español en Ciencias de la Salud (IBECS) a través de la Biblioteca Virtual en Salud (BVS). Para contemplar los estudios de literatura gris, también se utilizó la Biblioteca Digital Brasileña de Tesis y Disertaciones (BDTD) de la Coordinación de Perfeccionamiento del Personal de Nivel Superior (CAPES).

Los términos relacionados con el acrónimo PCC se adaptaron a cada plataforma de datos, teniendo en cuenta las variaciones y los operadores booleanos (AND y OR) para desarrollar las estrategias finales, que se presentan en el Cuadro 1.

**Cuadro 1:** Sintaxis de construcción, descriptores/palabras clave y operadores booleanos utilizados en las bases de datos – Teresina, PI, Brasil, 2024

Base de datos	Estrategia de búsqueda
PubMed^®^ via NLM N = 1210	((“health of the elderly”[All Fields]) OR (“aged”[MeSH Terms]) OR (“health services for the aged”[MeSH Terms]) OR (“aged, 80 and over”[MeSH Terms]) OR (“hospitalization”[MeSH Terms])) AND ((“pressure ulcer”[MeSH Terms]) OR (“risk assessment”[MeSH Terms]))) AND ((“nursing”[MeSH Terms]) OR (“nursing, team”[MeSH Terms]) OR (“nursing care”[MeSH Terms]) OR (“nurses improving care for health system elders”[MeSH Terms]))
Web of Science™ Colección Principal N = 1068	ALL = ((Aged* OR “Health of the Elderly” OR “Health Services for the Aged” OR “Health of the Elderly” OR “Aged, 80 and over” OR Hospitalization*) AND (Pressure Ulcer* OR “Risk Assessment”) AND (Nursing* OR Nursing, Team* OR “Nursing Care” OR “Nurses Improving Care for Health System Elders”))
Scopus^®^ N = 622	((Aged* OR “Health of the Elderly” OR “Health Services for the Aged” OR “Health of the Elderly” OR “Aged, 80 and over” OR Hospitalization*) AND (Pressure Ulcer* OR “Risk Assessment”) AND (Nursing* OR Nursing, Team* OR “Nursing Care” OR “Nurses Improving Care for Health System Elders”))
Embase^®^ N = 1688	(aged* OR ‘health services for the aged’/exp OR ‘health services for the aged’ OR ‘health of the elderly’ OR ‘aged, 80 and over’/exp OR ‘aged, 80 and over’ OR hospitalization*) AND ((‘pressure’/exp OR pressure) AND ulcer* OR ‘risk assessment’/exp OR ‘risk assessment’) AND ((nursing* OR ‘nursing,’/exp OR nursing,) AND team* OR ‘nursing care’/exp OR ‘nursing care’ OR ‘nurses improving care for health system elders’/exp OR ‘nurses improving care for health system elders’) AND [embase]/lim
BDENF, LILACS e IBECS via BVS N = 278	((“Saúde do Idoso”) OR (idos*) OR (“Serviços de Saúde para Idosos”) OR (“Saúde do Idoso”) OR (“Idoso de 80 Anos ou mais”) OR (Hospitalizaç*)) AND ((“Úlcera por Pressão”) OR (“Medição de Risco”)) AND ((“Enfermagem”) OR (“Equipe de Enfermagem”) OR (“Cuidados de Enfermagem”) OR (“Cuidado de Enfermagem ao Idoso Hospitalizado”))
BDTD de CAPES N = 50	(“Saúde do Idoso” OR idoso OR “Serviços de Saúde para Idosos” OR “Saúde do Idoso” OR “Idoso de 80 Anos ou mais” OR Hospitalização) AND (“Úlcera por Pressão” OR “Medição de Risco”) AND (“Enfermagem” OR “Equipe de Enfermagem” OR “Cuidados de Enfermagem” OR “Cuidado de Enfermagem ao Idoso Hospitalizado”)

Las búsquedas se realizaron el 17 de junio de 2024, utilizando el acceso remoto a las bases de datos a través del portal de revistas de la Coordinación de Perfeccionamiento del Personal de Nivel Superior (CAPES), al que se accedió a través de la Comunidad Académica Federada (CAFe) con el inicio de sesión de la Universidad Federal de Piauí (UFPI).

### Selección de la Fuente de Evidencia

La selección del material relacionado con el tema se realizó con la ayuda del sistema de gestión de referencias Rayyan CQRI Systems^(17)^. Inicialmente, los archivos que contenían la bibliografía encontrada en cada fuente de información se exportaron al sistema. Dos revisores, de forma independiente, llevaron a cabo la exclusión del material duplicado. A continuación, procedieron a la lectura de los resúmenes de los textos restantes, atribuyendo conceptos de aceptación o rechazo según los criterios de inclusión y exclusión establecidos. El proceso se llevó a cabo de forma ciega, utilizando el recurso*blind on*puesto a disposición por el sistema, y cualquier discrepancia entre los revisores fue resuelta por un tercer revisor.

Tras esta etapa, los textos seleccionados se leyeron íntegramente y se evaluaron según los criterios de inclusión y exclusión. Los textos aceptados se utilizaron para la extracción de datos. Los resultados de este proceso se organizaron en un diagrama que presenta las fases de identificación, selección, inclusión y selección de los textos para la revisión.

### Extracción de Datos

La extracción de datos para su inclusión en esta revisión se realizó a partir de la lista de referencias generada en Rayyan, que se exportó a una hoja de cálculo de Microsoft Excel Posteriormente, los tres revisores completaron la hoja de cálculo de forma independiente, siguiendo un formulario elaborado específicamente para esta etapa, con el objetivo de cumplir con el propósito y la cuestión de la revisión. Durante la fase de protocolo, se desarrolló y probó un borrador de tabla para registrar la información principal. Este borrador se perfeccionó a lo largo de la fase de revisión y dio lugar a la versión final, que incluyó los siguientes datos extraídos de cada fuente: fuente de información, autor(es), título, tipo, método, país, idioma, descriptores/palabras clave y estrategias de prevención de LP en personas mayores realizadas por el equipo de enfermería.

### Presentación de los Resultados

Los hallazgos se resumieron utilizando el método de reducción de datos, mediante la lectura crítica y la clasificación de los resultados en categorías conceptuales^(18)^. Los resultados se presentaron en figuras y cuadros. El primer cuadro detallaba la información descriptiva de los estudios, incluyendo la fuente de información, autor(es), título, tipo, método, país, idioma y descriptores/palabras clave. El segundo cuadro sintetizó las estrategias de prevención de LP en personas mayores hospitalizadas que pueden ser aplicadas por el equipo de enfermería.

La Figura 1 presenta el proceso de identificación, selección e inclusión de los artículos. La Figura 2, un diagrama circular, organiza las estrategias de prevención de LP en personas mayores hospitalizadas identificadas en la revisión. A diferencia de una jerarquía, el diagrama muestra las estrategias de forma interdependiente, con intervenciones posicionadas en segmentos que evidencian su complementariedad y relevancia igualitaria. Este formato favorece una visualización integrada de las acciones, destacando la contribución de cada estrategia para la prevención eficaz de las úlceras por presión en el contexto de las personas mayores hospitalizadas.

**Figura 1:** Flujograma del proceso de identificación, selección e inclusión de los artículos – Teresina, PI, Brasil, 2024.

Fuente: Adaptado de Page *et al.* (2021)^(16)^.

**Figura 2:** Diagrama circular de las estrategias de prevención de lesiones por presión en personas mayores realizadas por el equipo de enfermería – Teresina, PI, Brasil, 2024.

La discusión se basó en la literatura seleccionada y en otros estudios científicos relacionados con el tema. Además, se utilizaron documentos de entidades que promueven la creación y difusión de directrices basadas en la evidencia, con el objetivo de educar y capacitar a los profesionales de la salud, además de desarrollar políticas orientadas a mejorar la prevención y el tratamiento de las úlceras por presión.

### Aspectos Éticos

Al tratarse de una revisión de alcance sin participación directa de seres humanos, no fue necesario someterla al Comité de Ética en Investigación, de acuerdo con las directrices del JBI. Todas las etapas se llevaron a cabo de conformidad con los principios éticos aplicables a la investigación, garantizando la transparencia, el rigor metodológico y la integridad científica. Se respetaron los derechos de autor y se citaron correctamente las fuentes, garantizando la reproducibilidad y la fiabilidad de los resultados.

## RESULTADOS

Las estrategias de búsqueda identificaron un total de 4867 publicaciones. Tras eliminar 412 duplicados, quedaron 4455 estudios para la selección de títulos y resúmenes. De estos, se seleccionaron 97 para su lectura completa. Durante esta fase, no se pudieron recuperar 13 estudios, lo que dio como resultado 84 artículos que se leyeron en su totalidad. Tras un análisis minucioso, se excluyeron 73 artículos, quedando 11 estudios para componer esta revisión. Se documentaron todos los motivos de exclusión con el fin de elaborar el informe de exclusiones para su posterior exposición. Es importante destacar que no fue posible identificar estudios adicionales por otros métodos, por lo que no se añadieron más pruebas a la presente revisión (Figura 1).

A partir del análisis de los 11 estudios incluidos, se observó que la revista con mayor número de publicaciones sobre el tema fue la International Wound Journal, con dos publicaciones (18,18%). Además, los estudios fueron realizados por 36 autores diferentes. Los años con mayor número de publicaciones fueron 2021, 2019 y 2015, cada uno con dos publicaciones (18,18%), mientras que los demás años registraron una publicación (9,09%) cada uno. En cuanto a la tipología, se observó que todos los estudios analizados eran artículos científicos, siendo nueve investigaciones de campo (81,82%). El método más utilizado fue el cuasi-experimental, presente en tres estudios (27,27%). Además, China fue el país de origen más representativo, con tres estudios (27,27%).

En cuanto al idioma, la mayoría de los textos estaban disponibles en inglés (72,73%). Por último, las palabras clave destacaron las estrategias de prevención y tratamiento de la LP en personas mayores, enfatizando la importancia de los cuidados de enfermería continuos, la terapia nutricional y el uso de tecnología para la retroalimentación en tiempo real. Esta información se detalla en la Cuadro 2.

**Cuadro 2:** Fuentes de información seleccionadas – Teresina, PI, Brasil, 2024.

No.	Fuente y año	Autor	Título	País/idioma	Palabras clave	Método	Resultados
01	*Int. Wound J.* 2024	Deng et al.^(19)^	*Effects of predictive nursing interventions on pressure ulcer in elderly bedridden patients*	China/Inglés	*Bedridden; Efficacy; Elderly;* *Predictive Nursing; Pressure Ulcers*	Cuasiexperimental, comparación entre el grupo de control (cuidados convencionales) y el grupo de intervención (enfermería predictiva), n = 120.	La incidencia de LP fue significativamente menor en el grupo de intervención (10%) en comparación con el grupo de control (38,3%) (p < 0,001). Las puntuaciones de la escala de Braden fueron superiores en el grupo de intervención (p < 0,001). El tiempo transcurrido hasta el inicio de la LP fue mayor en el grupo de intervención (14,57 ± 3,21 días frente a 6,25 ± 2,36 días, p < 0,001). Las puntuaciones de ansiedad (SAS) y depresión (SDS) fueron menores en el grupo de intervención tras la atención (p < 0,001). La satisfacción de los pacientes fue mayor en el grupo de intervención (98,3% frente a 85%, p = 0,008).
02	*Am. J. Transl. Res.* 2021	Xiao et al.^(20)^	*The preventive effect of seamless nursing care on pressure ulcer and related complications in elderly inpatients*	China/Inglés	*Seamless Nursing Care; Elderly Inpatients; Pressure Ulcer; Complications*	Ensayo clínico aleatorizado con 132 ancianos hospitalizados (≥65 años), divididos en un grupo de control (cuidados convencionales, n = 66) y un grupo de intervención (cuidados convencionales + enfermería sin costuras, n = 66).	La incidencia de LP fue menor en el grupo de intervención (3,03%) en comparación con el grupo de control (21,21%) (p = 0,001). La tasa de complicaciones fue menor en el grupo de intervención (9,09% frente a 33,33%, p < 0,05). El tiempo medio de hospitalización se redujo en el grupo de intervención (21,34 ± 4,38 frente a 26,24 ± 6,11 días, p < 0,001). La satisfacción de los pacientes fue mayor en el grupo de intervención (83,33% frente a 59,09%, p = 0,002).
03	*Rev. Esc. Enferm.* 2021	Morrudo Garcia et al.^(21)^	Diagnóstico de enfermagem em pessoa idosa com risco para lesão por pressão	Brasil/Portugués	*Idoso; Hospitalização; Lesão por Pressão; Diagnóstico de Enfermagem; Cuidados de Enfermagem*	Se trata de un estudio exploratorio, descriptivo, transversal, cuantitativo, realizado con 87 historias clínicas de ancianos hospitalizados en una unidad de clínica médica de un hospital universitario de Brasil.	24 ancianos (27,6%) presentaban riesgo de LP. Factores asociados: estar encamado, restricción de posicionamiento en la cama y necesidad de ayuda a la locomoción (p < 0,05). Intervenciones predominantes: movilidad, control de la presión, supervisión de la piel, nutrición e higiene.
04	*Int. J. Clin. Exp. Med.* 2020	Yu et al.^(22)^	*Analysis of the effect of comprehensive nursing on pressure ulcers risk and psychological state in severe elderly patients*	China/Inglés	*Pressure Ulcers; Elderly; Severe; Psychological State; Comprehensive Nursing*	Estudio retrospectivo en el que se compararon 103 ancianos graves antes (grupo de control, 2017) y 103 después (grupo de intervención, 2018) de la implementación en de cuidados integrales de enfermería.	La incidencia de LP fue significativamente menor en el grupo de intervención (11,65%) en comparación con el control (39,81%) (p < 0,05). El tiempo medio transcurrido hasta la formación de la LP fue menor en el grupo de intervención (4,53 ± 2,81 días frente a 10,81 ± 3,56 días, p < 0,05). La satisfacción de los pacientes fue mayor en el grupo de intervención (91,14% frente a 76,67%, p < 0,05).
05	*Int Wound J.* 2019	Eglseer et al.^(23)^	*Nutritional management of older hospitalised patients with pressure injuries*	Austria/Español	*Hospital; Malnutrition; Nutrition Therapy; Pressure Injury; Pressure Ulcer*	Estudio transversal multicéntrico en 33 hospitales de Austria con 1412 pacientes hospitalizados ≥70 años. Evaluó las intervenciones nutricionales, el riesgo de LP y el estado nutricional.	El 48% de los pacientes estaban en riesgo de desarrollar LP y el 5% tenían LP. Las intervenciones nutricionales más frecuentes: soporte alimentario (50,7%), cribado nutricional (39,4%). Sólo el 28,2% de los pacientes con LP fueron derivados a un nutricionista.
06	*J. Wound Ostomy Continence Nurs.* 2019	Hultin et al.^(24)^	*Information and communication technology can increase patient participation in pressure injury prevention: a qualitative study in older orthopedic patients*	Suecia/Inglés	*Information and Communication Technology; Older Patients; Patient Participation; Pressure Injuries; Pressure Injury Prevention; Real-Time Feedback*	Estudio cualitativo en una unidad de rehabilitación ortopédica en Suecia con 31 pacientes ≥65 años de edad. Datos recogidos mediante entrevistas semiestructuradas analizadas mediante análisis de contenido.	La mayoría de los pacientes (n = 21) fueron capaces de utilizar *el mapeo continuo de la presión a pie de cama* (CBPM). Los participantes informaron de un mayor conocimiento de la prevención de las lesiones por presión (LP) y empezaron a recolocarse con más frecuencia. Los principales beneficios fueron el aumento de la independencia en el autocuidado y la participación activa en la prevención de las lesiones por presión. El CBPM proporcionó información visual en tiempo real, permitiendo ajustes inmediatos de las posiciones de los pacientes para reducir la presión en zonas vulnerables. La tecnología resultó ser útil para prevenir la LP y podría integrarse en la atención clínica como una característica estándar.
07	*Appl. Nurs. Res.* 2016	Bååth et al.^(25)^	*Prevention of heel pressure ulcers among older patients – from ambulance care to hospital discharge: A multi-centre randomized controlled trial*	Suecia/Inglés	*Heel Pressure Ulcer; Prevention; Ambulance Care; Emergency Department; Acute Care Delivery Chain; RCT*	Ensayo controlado aleatorio multicéntrico con 183 ancianos (≥70 años) transportados en ambulancia. El grupo de intervención utilizó un dispositivo de suspensión del talón.	La incidencia de LP en el talón fue significativamente menor en el grupo de intervención (14,6%) en comparación con el control (30%) (p = 0,017). El dolor asociado a las lesiones fue menor en el grupo de intervención. El dispositivo de suspensión del talón redujo el tiempo de contacto directo de la piel con las superficies de apoyo, minimizando la formación de LP. Los resultados sugieren que el uso de dispositivos tecnológicos, como los soportes de redistribución de la presión, debería incorporarse de forma rutinaria a la atención prehospitalaria.
08	*Enfermería. Stand.* 2015	Barry y Nugent^(26)^	*Pressure ulcer prevention in frail older people*	Irlanda/Inglés	*Education; Frail Older People; Incidence; Older People; Pressure Ulcers;* *Pressure Ulcer Prevention; Prevalence; Wound Care; Wound Management*	Revisión narrativa sobre la prevención de las LP en personas mayores frágiles, abordando programas educativos, liderazgo clínico y trabajo multidisciplinar.	Los programas educativos estructurados redujeron la incidencia de la LP en personas mayores frágiles en un 73%. El enfoque SSKIN (Surface, Skin Inspection, Movement, Incontinence, Nutrition) demostró su eficacia en la prevención y la estandarización de los cuidados. El estudio refuerza la necesidad de utilizar tecnologías de control de la presión y superficies especializadas para optimizar la prevención de la LP.
09	*Index Enferm.* 2015	Sousa et al.^(27)^	*Concepciones teóricas de Neuman asociadas con la prevención de las úlceras por presión: Un estudio de caso*	Brasil/Español	*Úlcera por Presión; Teoría de Enfermería; Cuidado de Enfermería.*	Reporte de caso basado en la Teoría de Sistemas de Betty Neuman para la prevención de las LP. Caso de una anciana hospitalizada durante 64 días en la Unidad de Cuidados Intensivos (UCI).	La paciente no desarrolló LP durante su hospitalización, lo que sugiere la eficacia de las estrategias preventivas basadas en la teoría de Neuman.
10	*Crit. Care Nurs. Clin. North Am.* 2007	Stotts y Wu^(28)^	*Hospital recovery is facilitated by prevention of pressure ulcers in older adults*	EE.UU./Inglés	Sem palavras-chave	Revisión narrativa sobre la prevención de las LP en personas mayores hospitalizadas y su impacto en la recuperación.	La incidencia de LP en personas mayores hospitalizadas oscila entre el 0,4% y el 38%. La prevención se asocia con estancias hospitalarias más cortas y mejores resultados funcionales.
11	*Enfermería Geriátrica.* 1997	Maklebust^(29)^	*Pressure ulcers: decreasing the risk for older adults*	EE.UU./Inglés	Sem palavras-chave	Revisión narrativa sobre los factores de riesgo y las estrategias de prevención de las LP en los adultos mayores.	Los principales factores de riesgo incluyen la inmovilidad, la humedad excesiva, la fricción, el cizallamiento y la desnutrición. Las medidas preventivas incluyen el reposicionamiento, las superficies de apoyo y una nutrición adecuada.

El análisis de estrategias para prevenir la LP en ancianos hospitalizados reveló 10 enfoques importantes que puede llevar a cabo el equipo de enfermería: evaluación inicial del riesgo; reevaluación y monitorización continuas; redistribución de la presión; mantenimiento de la integridad de la piel; tratamiento de la LP, apoyo nutricional e hídrico; educación e implicación de pacientes y familiares; formación del equipo sanitario, aplicación de instrumentos estandarizados y uso de tecnología (Cuadro 3).

**Cuadro 3:** Estrategias de prevención de lesiones por presión en ancianos que pueden ser realizadas por el equipo de enfermería – Teresina, PI, Brasil, 2024.

No.	Estrategia	Estrategia Descripción
01	Evaluación inicial del riesgo	Realizar una evaluación inicial con escalas de riesgo, como Braden y Norton, para identificar a los pacientes con alto riesgo de LP. Documentar y actualizar periódicamente la evaluación para reflejar los cambios en el estado del paciente^(19,20,25–28)^.
02	Reevaluación y monitorización continuas	Inspeccionar diariamente la piel para detectar signos precoces de LP, como cambios de color o integridad. Documentar los hallazgos, aplicar intervenciones inmediatas cuando sea necesario y ajustar el plan de cuidados en func^(20-22,24–29)^.
03	Redistribución de la presión	Adoptar técnicas de redistribución de la presión, como cambios frecuentes de decúbito y el uso de superficies de apoyo especializadas, incluidos colchones de espuma de alta densidad, colchones alternantes y cojines de gel o espuma. Registrar la eficacia de estas intervenciones para prevenir las complicaciones asociadas a las prominencias óseas^(19–22,24–29)^.
04	Mantener la integridad de la piel	Asegurarse de que la piel del paciente está limpia y seca. Aplicar barreras protectoras en las zonas vulnerables a la humedad (incontinencia urinaria o fecal) y utilizar humectantes para evitar la sequedad. Controlar periódicamente la integridad de la piel y ajustar los cuidados según sea necesario^(21,22,26–29)^.
05	Tratamiento de las lesiones por presión	Limpiar y tratar las heridas por LP según los protocolos establecidos, incluyendo el uso de analgésicos y tratamiento antiinfeccioso cuando sea necesario. Controlar la evolución de las heridas y ajustar el tratamiento según ellas^(20)^.
06	Apoyo nutricional e hídrico	Identificar las preferencias alimentarias, detectar la malnutrición y vigilar el estado nutricional. Aplicar suplementos proteicos o nutrición enteral/parenteral cuando esté indicado. Fomentar la ingesta de líquidos y alimentos, ajustando la consistencia para facilitar la ingestión, según los protocolos nutricionales recomendados^(21–23,25–29)^.
07	Educación y participación del paciente y la familia	Incluir a pacientes y familiares en el plan de cuidados para la prevención de la LP. Proporcionar una orientación clara sobre los cuidados necesarios, los signos de alarma y las medidas preventivas. Asegurarse de que comprenden y son capaces de aplicar las directrices^(19,20,22,24,28)^.
08	Formación del equipo sanitario	Desarrollar programas de formación continuada para el equipo sanitario sobre evaluación de riesgos, intervenciones preventivas y gestión de la PL. Asegurarse de que el equipo está al día de las mejores prácticas y protocolos^(22,26)^.
09	Aplicación de instrumentos normalizados	Aplicar protocolos, listas de comprobación o instrumentos normalizados, como el SSKIN *Care Bundle, la Malnutrition Universal Screening Tool* (MUST) y la Safety Cross, para garantizar un enfoque sistemático y coherente de la prevención de la LP^(23,25–28)^.
10	Uso de la tecnología	Utilizar sistemas tecnológicos avanzados, como *el mapeo continuo de la presión a pie de cama* (*Continuous Bedside Pressure Mapping* - CBPM), para monitorizar continuamente el riesgo de LP. Integrar los datos tecnológicos en las prácticas asistenciales para optimizar la prevención de lesiones y la respuesta^(24)^.

Para facilitar la comprensión de las estrategias de prevención de la LP en personas mayores hospitalizadas, las intervenciones se han organizado en un modelo circular que presenta cuatro áreas interrelacionadas, cada una de las cuales representa aspectos clave de los cuidados necesarios (Figura 2).

El área de “Evaluación e identificación de riesgos” representa la base de las intervenciones preventivas. Incluye la evaluación inicial del riesgo, esencial para identificar a los pacientes vulnerables, y la reevaluación continua, que permite controlar el estado del paciente a lo largo del tiempo.

A continuación, el área “Intervenciones prácticas directas” abarca acciones específicas, como la redistribución de la presión, el mantenimiento de la integridad de la piel y el tratamiento de las lesiones establecidas. Estas prácticas pretenden prevenir la aparición de nuevas lesiones y minimizar el daño de las ya existentes.

El área “Apoyo adicional y formación” hace hincapié en la importancia de proporcionar apoyo nutricional e hídrico, promover la educación e implicación del paciente y la familia, y formar al equipo sanitario. Estas medidas aumentan la eficacia de las intervenciones preventivas y refuerzan la colaboración asistencial.

Por último, el área “Instrumentos y tecnología” reúne los elementos más avanzados de la asistencia, incluida la aplicación de instrumentos normalizados y el uso de tecnologías para mejorar la eficacia y la precisión de las intervenciones preventivas.

## DISCUSIÓN

Los resultados de este estudio permitieron agrupar en diez categorías las estrategias de prevención de LP adoptadas por el equipo de enfermería en personas mayores hospitalizadas. Estas recomendaciones están en consonancia con las directrices internacionales de la*Prevention and Treatment of Pressure Ulcers/Injuries: Quick Reference Guide*2019, que, aunque son generales, orientan la adaptación de las prácticas preventivas al contexto hospitalario y a la población de edad avanzada^(30)^.

La evaluación sistemática de la piel es una medida esencial para la prevención. La literatura recomienda una inspección holística, de la cabeza a los pies, con especial atención a las prominencias óseas y a las áreas bajo los dispositivos, con el fin de identificar precozmente los signos de lesión. Esta evaluación debe ser periódica y tener en cuenta las superficies de apoyo, la integridad de la piel, la movilidad, la comodidad y el nivel de actividad, a fin de respaldar las intervenciones adecuadas^(31)^.

Las evidencias indican que las intervenciones predictivas de enfermería son más eficaces que los enfoques convencionales en la prevención de las úlceras por presión en personas mayores encamadas durante largos periodos, lo que aumenta la satisfacción de los pacientes y presenta una alta viabilidad^(19)^. Las escalas de Braden (1987) y Norton (1962) son herramientas ampliamente utilizadas para estratificar el riesgo de LP, al analizar factores como la movilidad, la humedad, la sensibilidad, la nutrición y la fricción, lo que favorece la implementación de medidas preventivas específicas^(32,33)^.

La reevaluación periódica y el monitoreo continuo potencian estos resultados. Uno de los estudios incluidos mostró que el uso de botas con dispositivo de suspensión del talón en pacientes mayores de 70 años redujo la incidencia de LP cuando el riesgo se evaluó desde la atención prehospitalaria hasta el alta, garantizando la continuidad de la atención^(27)^.

Así, las estrategias de evaluación inicial, reevaluación y monitoreo, comprendidas en la categoría “Evaluación e identificación de riesgos”, presentan un bajo costo, una amplia aplicabilidad y una alta eficacia para la identificación temprana de factores de riesgo y para la activación oportuna de intervenciones preventivas.

La redistribución de la presión, mediante el cambio de decúbito y el uso de superficies de apoyo, es una estrategia esencial para la prevención de las úlceras por presión. Sin embargo, una revisión sistemática señala la falta de consenso sobre los intervalos ideales de reposicionamiento, que varían entre 2, 3, 4 y 6 horas^(34)^. Por lo tanto, las directrices de la*Wound Ostomy & Continence Nurses Society*recomiendan ajustar la frecuencia del reposicionamiento a las condiciones clínicas y las necesidades individuales, definiendo este cuidado como estándar^(35)^.

Las superficies de apoyo contribuyen a aliviar la presión en las zonas vulnerables y pueden clasificarse en sistémicas y locales. Las primeras incluyen colchones de alta especificación, colchones de aire reactivos o de presión alterna y otros dispositivos avanzados, ampliamente utilizados para proteger regiones como la sacrococcígea, más susceptible al desarrollo de LP. Las superficies locales, como las almohadillas de gel o de aire, se adaptan a las especificidades anatómicas. Los dispositivos antiguos, como los colchones y almohadillas de agua, no se recomiendan debido a su eficacia limitada y a los riesgos asociados a su uso inadecuado^(30,36)^.

Aunque estudios más antiguos ya destacaban la importancia de la redistribución de la presión^(29)^, las directrices actuales refuerzan prácticas actualizadas, como la posición lateral a 30° y el uso de dispositivos de suspensión para los talones, junto con planes individualizados que concilian el riesgo, la comodidad y las tecnologías avanzadas^(29,30)^.

El mantenimiento de la integridad de la piel es otro componente fundamental. En entornos con alta prevalencia de xerosis cutánea, dermatitis asociada a la incontinencia, laceraciones e intertrigo, las inspecciones frecuentes y el mantenimiento de la piel seca son medidas esenciales. Las evidencias demuestran que los programas de cuidados estructurados, que incluyen limpiadores suaves y productos sin enjuague, son eficaces para prevenir daños, especialmente en personas mayores y pacientes pediátricos^(37)^.

En uno de los estudios analizados, el uso de diagnósticos y planes de cuidado específicos para personas mayores contribuyó a la prevención y a la mejora de la calidad del cuidado, con intervenciones centradas en la higiene, el manejo de la incontinencia, el control de la piel y la nutrición, el posicionamiento y la supervisión de la presión sobre las áreas vulnerables^(21)^.

Aunque el enfoque es la prevención, el tratamiento de las úlceras por presión es parte integrante de la atención. Incluye una limpieza adecuada, el uso de apósitos y técnicas terapéuticas para acelerar la cicatrización y prevenir complicaciones. El desbridamiento —quirúrgico, autolítico, enzimático o mecánico— es esencial para eliminar el tejido necrótico, y el control de la infección requiere el uso de antimicrobianos tópicos o sistémicos, manteniendo la lesión limpia y cubierta para reducir los riesgos y favorecer la cicatrización^(38)^.

Un estudio demostró que la atención de enfermería continua redujo las complicaciones y aumentó la satisfacción de los pacientes ancianos hospitalizados, integrando la prevención, el tratamiento y el apoyo físico y psicológico, con la participación activa de los pacientes y sus familiares^(20)^.

En el diagrama circular, las estrategias de redistribución de la presión, mantenimiento de la integridad de la piel y tratamiento de las LP componen el área “Intervenciones prácticas directas”, fundamentales para prevenir nuevas lesiones y favorecer la recuperación de las existentes mediante cuidados especializados.

En el ámbito del área “Apoyo adicional y capacitación”, la nutrición y la hidratación adecuadas son componentes esenciales para la prevención y la cicatrización de las LP. El equipo de enfermería debe monitorear el estado nutricional y fomentar la ingesta de líquidos, ya que la desnutrición y la deshidratación aumentan la morbimortalidad y comprometen la cicatrización. La pérdida de peso no planificada y la baja ingesta de líquidos son factores de riesgo que requieren una identificación temprana y un manejo oportuno^(39)^.

Las directrices actuales recomiendan un enfoque multidisciplinario para el manejo de las úlceras por presión^(30)^. Los nutricionistas y dietistas ajustan la dieta e indican suplementos proteicos de acuerdo con las necesidades individuales, optimizando el estado nutricional y favoreciendo la prevención y la cicatrización^(40)^. Sin embargo, aún hay poca evidencia sobre la eficacia de composiciones nutricionales específicas^(41)^. Un estudio transversal y multicéntrico con pacientes ≥70 años en riesgo de LP evidenció que la atención nutricional ofrecida está por debajo de lo recomendado: el cribado nutricional, la derivación al nutricionista y la suplementación proteica son prácticas poco frecuentes en la rutina asistencial^(23)^.

La participación de los pacientes y sus familiares en el plan de cuidados es otro pilar de esta área, que abarca orientaciones sobre señales de alerta y medidas preventivas. Sin embargo, esta estrategia se implementa poco debido a la falta de materiales educativos adecuados; cuando están disponibles, suelen ser incompletos, descuidando las directrices para los cuidadores y los recursos visuales^(42)^. Uno de los estudios analizados refuerza la importancia de integrar la prevención de la LP en la atención global de las personas mayores hospitalizadas, destacando la actuación de equipos interdisciplinarios de evaluación geriátrica^(28)^.

La capacitación del equipo es decisiva para la educación del paciente y la calidad de la atención. Los programas de educación continua, presenciales o a distancia, deben estar estructurados, supervisados y utilizar metodologías activas, como la simulación y la enseñanza en línea. Las evidencias indican que las capacitaciones de entre 45 minutos y dos horas, incluso en formato de aprendizaje electrónico, son eficaces para mejorar la identificación temprana de riesgos y la implementación de medidas preventivas^(43–45)^.

En un estudio retrospectivo realizado en el Segundo Hospital Afiliado de la Universidad Médica de Wenzhou, 103 ancianos hospitalizados fueron atendidos por enfermeros recién capacitados sobre las causas, la prevención y el tratamiento de la LP. La atención integral redujo significativamente el riesgo de LP, mejoró el estado psicológico y la calidad de vida de los pacientes y aumentó la comodidad durante la hospitalización^(22)^.

En el modelo circular, las estrategias de apoyo nutricional e hídrico, asociadas a la educación y la participación de los pacientes y sus familiares, conforman el área “Apoyo adicional y capacitación”. Estas medidas son fundamentales para la prevención de la PL en personas mayores hospitalizadas, una población especialmente susceptible a la desnutrición y la deshidratación, y requieren una atención integral, interdisciplinaria y continua.

En el área “Instrumentos y tecnología” del diagrama circular, la implementación de protocolos estructurados es un eje central para la prevención de las LP. El cumplimiento riguroso de protocolos bien definidos mejora la calidad de la atención y reduce significativamente la incidencia de estas lesiones. Los programas específicos potencian su eficacia al garantizar una atención integral y continua al paciente^(46)^. En este proceso, la enfermería desempeña un papel central en la aplicación, el seguimiento y el ajuste de las medidas, adaptándolas a las necesidades individuales^(47)^.

La revisión identificó herramientas esenciales para la prevención de las LP. El SSKIN*Care Bundle*es un enfoque integral basado en cinco pilares: superficies de apoyo adecuadas, supervisión regular de la piel, control de la humedad, nutrición y movimiento del paciente. La Herramienta Universal de Detección de Malnutrición (MUST) evalúa el riesgo de malnutrición mediante factores como el índice de masa corporal (IMC), la pérdida de peso reciente y el impacto de la enfermedad en el estado nutricional. Por su parte, la Cruz de Seguridad o Calendario de Seguridad es una estrategia visual utilizada en las unidades de salud para monitorear eventos adversos y promover un entorno de cuidado más seguro^(26)^.

Las tecnologías avanzadas han demostrado ser eficaces como estrategias de prevención. Los métodos de aprendizaje automático para modelos predictivos, reconocimiento de postura y análisis de imágenes contribuyen a la detección temprana y al monitoreo continuo^(48)^. Los sensores portátiles ayudan a cumplir con los protocolos de reposicionamiento, reduciendo la incidencia de úlceras por presión^(49)^. Otras tecnologías, como el ultrasonido, la termografía, la medición de la humedad subepidérmica y la espectroscopia láser Doppler, superan las limitaciones de la inspección visual tradicional, lo que permite un enfoque más preciso y proactivo^(50)^.

A pesar de estos avances, la implementación tecnológica en los hospitales, especialmente en los países en desarrollo, se enfrenta a retos: falta de infraestructura, altos costos, necesidad de capacitación continua, impacto presupuestario, desigualdad en el acceso y dependencia de equipos importados. Estos factores refuerzan la importancia de estrategias adaptadas a la realidad local, que busquen la accesibilidad y la sostenibilidad^(51)^.

Un estudio incluido en esta revisión evaluó el uso del Continuous Bedside Pressure Mapping (CBPM) en una unidad de rehabilitación ortopédica en Uppsala, Suecia, con pacientes mayores de 65 años. El CBPM utiliza una alfombra con sensores de presión conectados a un monitor, que indica en rojo las áreas de mayor presión. Esta visualización en tiempo real proporcionó información al equipo y a los pacientes, fomentando el cambio de posición y promoviendo el autocuidado. Tras la intervención, se observó una mejora en el conocimiento de los pacientes sobre los factores de riesgo y la prevención de las úlceras por presión, con un mayor compromiso con las medidas preventivas^(24)^.

En el modelo circular, la implementación de instrumentos estandarizados y el uso de tecnologías componen el área “Instrumentos y tecnología”. Estas estrategias requieren más recursos e implican intervenciones más complejas que las acciones directas de manejo, pero estructuran y califican las prácticas asistenciales, ofreciendo apoyo a las demás áreas, fortaleciendo la prevención secundaria y terciaria y contribuyendo a reducir los daños en las personas mayores hospitalizadas.

A pesar de la evidencia de la eficacia de las intervenciones puntuales, la prevalencia global de las lesiones por presión sigue siendo elevada. Esta paradoja se debe a barreras estructurales y organizativas, como la escasez de recursos humanos y materiales, la baja adhesión a las directrices, las lagunas de conocimientos y habilidades, la ausencia de una cultura de seguridad consolidada y las dificultades prácticas para incorporar las recomendaciones en la rutina asistencial. Incluso en hospitales con sistemas estructurados, un estudio reciente demuestra que la implementación de las directrices se ve obstaculizada por estos factores, lo que refuerza la idea de que las medidas aisladas son insuficientes sin cambios institucionales y políticas coherentes^(52)^.

Una limitación de esta revisión es la ausencia de una evaluación formal de la calidad metodológica de los estudios incluidos, lo que puede afectar a la solidez de las conclusiones. Esta limitación se atenuó mediante el análisis exhaustivo de la literatura científica y la consulta de las recomendaciones de organismos especializados. Aunque las revisiones de alcance no suelen realizar una evaluación crítica de los estudios, se trató de fundamentar las estrategias presentadas teniendo en cuenta las especificidades de la hospitalización de personas mayores, con recomendaciones más aplicables al contexto clínico.

Este estudio contribuye a la práctica de la enfermería al identificar evidencias que orientan la implementación de estrategias sistematizadas para la prevención de las úlceras por presión. Se destaca la necesidad de la capacitación continua del equipo, así como el uso racional de tecnologías para el monitoreo de riesgos y la evaluación de intervenciones, promoviendo una mayor seguridad y calidad en la atención. En el contexto hospitalario, estas estrategias deben adaptarse a los recursos y la infraestructura disponibles, especialmente en escenarios con limitaciones tecnológicas y de capacitación profesional. Por lo tanto, integrar estas acciones en los protocolos institucionales, invertir en educación continua y utilizar tecnologías emergentes de forma criteriosa son medidas esenciales para consolidar la prevención de las úlceras por presión como un estándar de atención seguro y eficaz.

Por último, los hallazgos de esta revisión están en consonancia con la literatura internacional, que enfatiza la necesidad de ampliar la investigación en enfermería sobre la prevención de las úlceras por presión, reforzando la importancia de las prácticas basadas en la evidencia para atender adecuadamente a esta población vulnerable^(53)^.

## CONCLUSIÓN

Las estrategias de prevención de las úlceras por presión identificadas en la revisión se organizaron en cuatro áreas: evaluación e identificación de riesgos, intervenciones prácticas directas, apoyo adicional y capacitación, e instrumentos y tecnología. Estas estrategias permiten al equipo de enfermería actuar de manera rápida y eficiente, empleando intervenciones integradas, optimizadas por la capacitación profesional y el uso de instrumentos estandarizados y tecnologías avanzadas.

Estos enfoques tienen implicaciones directas en la atención a las personas mayores. La evaluación temprana identifica los riesgos y permite una acción inmediata. Las intervenciones prácticas, como la redistribución de la presión y el cuidado de la piel, previenen el desarrollo de lesiones. El apoyo adicional, que incluye nutrición, educación y capacitación del personal, mejora la calidad de la atención. El uso de instrumentos estandarizados y tecnologías avanzadas facilita tanto la aplicación como el seguimiento de las prácticas preventivas. Cuando se aplican de manera integrada, estas estrategias garantizan una atención de calidad, reducen el riesgo de LP y promueven una atención integral y específica para las personas mayores hospitalizadas.

Se recomienda realizar estudios de intervención que exploren las diferentes estrategias, con especial atención a la implementación de tecnologías avanzadas y protocolos rigurosos. Estos estudios pueden reforzar la eficacia de las prácticas preventivas y promover mejoras en la calidad de la atención prestada a esta población vulnerable.

## Data Availability

Todo el conjunto de datos que respalda los resultados de este estudio fue publicado en el propio artículo.
